# Mechanism of ITGB2 in Osteoclast Differentiation in Osteoarthritis

**DOI:** 10.1111/cpr.70107

**Published:** 2025-07-29

**Authors:** Yang Yang, Rui Sun, Zhibin Lan, Qi Ma, Gang Wu, Di Xue, Zhirong Chen, Yajing Su, Ye Ma, Xiaolei Chen, Jiangbo Yan, Long Ma, Xiaoxin He, Kuanmin Tian, Xiaoyi Ma, Xue Lin, Qunhua Jin

**Affiliations:** ^1^ The Third Ward of Orthopaedic Department, Institute of Osteoarthropathy, Institute of Medical Sciences General Hospital of Ningxia Medical University Yinchuan People's Republic of China; ^2^ Ningxia Key Laboratory of Clinical and Pathogenic Microbiology General Hospital of Ningxia Medical University Yinchuan People's Republic of China

**Keywords:** integrin, osteoarthritis, osteoclast differentiation, Rac1, subchondral bone

## Abstract

Transcriptomics studies have identified integrin receptor β2 subunit (ITGB2) as a core gene in osteoarthritis (OA), strongly linked to osteoclast function in the subchondral bone. However, the mechanism through which ITGB2 regulates osteoclast function in OA remains unclear. In this study, we found that ITGB2 was negatively correlated with ITGB1 in the human subchondral bone. Proteomic analysis indicated that integrin binding is crucial in OA subchondral bone, with ITGB2 identified as a significantly upregulated protein in OA. In vitro experiments using immunoprecipitation and bimolecular fluorescence complementation revealed that ITGB2, but not ITGB1, directly interacts with Rac1 during osteoclast differentiation. Activated Rac1 promotes osteoclast differentiation and bone resorption through several mechanisms. ITGB2 knockdown reduced Rac1‐GTP levels and increased ITGB1 expression. ITGB2 inhibition reduced actin ring formation and microtubule migration to the cell edge during osteoclast differentiation. Additionally, overexpression of ITGB1 in ITGB2‐knockdown cells not only further suppressed ITGB2 expression but also exacerbated the inhibition of osteoclast differentiation. In a DMM mouse model, ITGB2 was associated with osteoclast activity in the subchondral bone. ITGB2 knockdown significantly reduced bone resorption and slowed OA progression by inhibiting osteoclastogenesis. In conclusion, our study identified a novel mechanism for the reciprocal regulation of integrin subunits. Moreover, inhibition of the ITGB2 signalling pathway slows subchondral bone remodelling in osteoarthritis by inhibiting osteoclast differentiation, offering a potential strategy for targeted therapeutic interventions.

## Graphical abstract

In osteoclast precursors, higher ITGB1 than ITGB2 expression increases ITGA2‐ITGB1 integrin. In OA, extracellular signals upregulate ITGB2 and downregulate ITGB1, enhancing ITGA2‐ITGB2 integrin. This activates Rac1 to Rac‐GTP, driving osteoclast differentiation. Hyperactive subchondral osteoclasts accelerate OA progression.

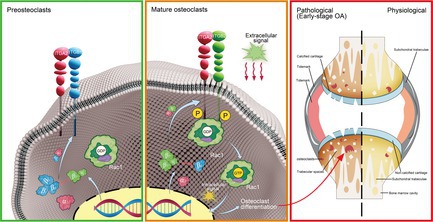

## 1 Introduction

Osteoarthritis (OA) is a common degenerative joint disease that is primarily characterised by cartilage degeneration, subchondral bone remodelling, and synovial inflammation [1, 2]. OA severely affects the quality of life of over 500 million people globally, approximately 7% of the population [3]. Currently, there is no effective treatment for OA. Existing treatments primarily aim to relieve symptoms rather than reverse disease progression [3, 4]. The aetiology of OA remains poorly understood. However, growing evidence indicates that imbalances in subchondral bone homeostasis are crucial in the onset and progression of osteoarthritis, potentially preceding cartilage damage [5].

Under physiological conditions, subchondral bone homeostasis is maintained by the closely coupled activities of osteoclasts (OCs) and osteoblasts (OBs), which dynamically regulate the mechanical forces applied to the joint [6]. However, in the early stages of osteoarthritis, abnormal mechanical stress promotes OC recruitment. Osteoclast overactivity results in the uncoupling of osteogenesis from OBs, triggering abnormal remodelling in the subchondral bone [7, 8]. Osteoclast formation and function involve a complex series of biological processes. Osteoclast precursors (OCPs) derive from bone marrow and are regulated during differentiation by various factors, including macrophage colony‐stimulating factor (M‐CSF) and receptor activator of nuclear factor κ‐B ligand (RANKL). M‐CSF promotes survival and proliferation of precursor cells, whereas RANKL induces their maturation into functional OCs. Numerous steps in osteoclast differentiation involve cell–cell interactions and cell‐matrix adhesion. These mechanisms not only contribute to osteoclast formation but also directly influence their activation and function [9].

Increasing evidence indicates that integrin‐mediated adhesion and signalling are essential in mechanotransduction and regulation of subchondral bone formation [10]. Integrins are heterodimeric transmembrane receptors composed of alpha (ITGA) and beta (ITGB) subunits. Integrins are well established as crucial mediators of OCs differentiation, proliferation, migration, and bone resorption [11] and are key in the pathogenesis of OA [12]. For example, ITGAV‐ITGB3 binds to colony‐stimulating factor‐1 receptors (cFms) to form the cytoskeleton necessary for OCs migration [13]. Additionally, the blockade of ITGA2‐ITGB1 inhibits IL‐7‐induced OCs differentiation and inflammatory bone resorption by diminishing IL‐17 production [14]. Integrin signalling is mediated by the activation of Rac1, a small GTPase. Rac1 regulates cytoskeletal reorganisation through its binding to integrins, thereby influencing cell migration and activation [15, 16]. Recent studies have indicated that RAC1 is essential for the differentiation of macrophages into OCs [17, 18]. ITGB2, a member of the integrin family, is widely expressed in leukocytes, particularly on monocytes [19]. Recent studies have indicated that ITGB2 is involved in OCs formation [20]. However, systematic studies on the specific function of ITGB2 in OCs development and its role in OA progression are lacking. These findings prompted us to explore the roles of integrin receptor subunits in OA.

In this study, we analysed transcriptomic data from the GEO database and proteomic data from clinical samples of 20 patients with OA. We found that ITGB2 levels were increased in patients with OA and were negatively correlated with ITGB1. Mechanistically, during subchondral bone formation, OCs express ITGA2, which binds to ITGB1 and ITGB2. ITGB2 promotes differentiation of bone marrow mesenchymal stem cells (MSCs) into OCs in OA by activating Rac1 and downregulating ITGB1. To further validate the significance of ITGB2 in OA development and its potential as a therapeutic target, we conducted experiments using an ITGB2 knockdown mouse model of OA. Our results confirmed that ITGB2 inhibits osteochondral bone remodelling in OA subchondral bone and is a potential therapeutic target for OA.

## 2 Materials and Methods

### 2.1 Human Samples

In this study, tibial plateau tissue samples (subchondral bone tissue) were obtained from 20 patients (aged 50–70 years) who underwent unilateral total knee arthroplasty at the General Hospital of Ningxia Medical University. The weight‐bearing area was designated the OA group, while the non‐weight‐bearing area on the same side served as the control group. Normal synovium samples were collected from age‐ and sex‐matched patients who underwent arthroscopic surgery for anterior cruciate ligament (ACL) injuries. Synovium from patients with OA was obtained from the same individuals who underwent total knee arthroplasty (TKA), as mentioned above. All patients were diagnosed with OA according to the criteria established by the American College of Rheumatology, and were excluded if they had secondary OA caused by connective tissue diseases, trauma, or metabolic disorders. Additionally, patients with systemic diseases that might affect joint pathology, such as diabetes or obesity, were excluded to ensure the homogeneity of the study population.

### 2.2 Extraction and Analysis of Gene Expression Data From GEO Database/Proteomics

This study analysed OA‐related gene data from the GEO database (https://www.ncbi.nlm.nih.gov/geo/) using synovial (GSE1919, GSE55235) and subchondral (GSE51588) bone samples. Differential expression analysis was conducted with the “limma” package in R. Weighted gene co‐expression network analysis (WGCNA) identified key gene modules, and candidate genes were derived from overlapping DEG and WGCNA results. Protein–protein interactions were assessed using STRING and Cytoscape, focusing on the top 10 genes. A nomogram model was developed with the “rms” package to predict osteoarthritis risk, evaluated by Harrell's C‐index and ROC curve analysis.

PRM analysis was performed on subchondral bone samples from 20 patients to compare protein expression between OA and control groups. Proteins were digested with trypsin and separated using high‐pH reverse‐phase chromatography. Liquid chromatography–tandem mass spectrometry (LC–MS/MS) was used to identify peptides with database matching for known proteins. Data‐independent acquisition (DIA) was used for protein quantification using the DIA software. Bioinformatic analysis identified differentially expressed proteins, whereas GO/KEGG analyses revealed pathways related to osteoarthritis.

### 2.3 Quantitative Assessment of Histology

The synovial membrane and subchondral bone tissues were collected from the knee joint and fixed in 4% paraformaldehyde for 24 h. Synovial tissue sections (4 μm) were prepared for haematoxylin–eosin (HE) staining to assess cell density and structure. Immunohistochemical analysis was performed to assess ITGB2 expression using a citrate buffer (cat. No. C9999, Sigma) for antigen retrieval and 3% hydrogen peroxide (cat. No. HX0645, Sigma) for blocking, followed by incubation with the primary antibody against ITGB2 (cat. No. ab307406, Abcam, UK, 1:100). DAB was used for the detection.

Subchondral bone sections were stained with HE staining and Safranin O‐fast green staining to identify the cartilage matrix distribution, and TRAP staining (cat. No. MK301; TaKaRa, Japan) was used to detect the OCs. ITGB2 immunohistochemistry in the subchondral bone was performed using steps similar to those used for the synovial tissue. For immunofluorescence, the frozen sections were fixed and permeabilised with 1% Triton X‐100 (cat. No. 9036‐19‐50, Sigma, USA). After blocking with 5% normal goat serum, the sections were incubated with ITGB2 (cat. No. ab307406, Abcam, UK, 1:100) and ITGB1 (cat. No. sc‐374429, Santa Cruz, USA, 1:100). Fluorescent secondary antibodies (Cat. No. A32766 and A32754; Thermo Fisher Scientific, USA, 1:10000) and DAPI for nuclear visualisation (cat. No. R37605 and R37606; Thermo Fisher Scientific, USA) were used to analyse protein expression and localisation.

### 2.4 Animal Samples

CRISPR/Cas9 was used to knockout ITGB2 in C57BL/6J GPT mice. A 20‐bp gRNA targeting ITGB2 was designed using the CRISPR Design Tool (MIT, http://crispr.mit.edu/) and mixed with Cas9 protein for microinjection into fertilised C57BL/6J Gpt eggs. The injected embryos were then transferred to pseudo‐pregnant females to generate F0 mice. DNA was extracted from tails to 5–7 days post‐birth for PCR and sequencing genotyping. F0 mice with confirmed mutations were mated with wild‐type C57BL/6J Gpt mice to produce F1 offspring, which were genotyped by PCR. Heterozygous F1 mice were selected for breeding to establish stable ITGB2 knockout inheritance.

In total, 60 healthy adult female C57BL/6 mice (age: 6 weeks; weight: 16 g) were acquired. A surgically induced osteoarthritis model was established in mice using the DMM (Destabilisation of the Medial Meniscus) method [21]. All surgical procedures were performed under pentobarbital anaesthesia to minimise animal discomfort and ensure safety. The procedure involved making an incision under sterile conditions to expose the medial meniscus, which was then detached to induce joint instability. Following surgery, the incision was sutured and the wound was disinfected daily to support recovery.

### 2.5 Cell Isolation, Culture, Treatment, siRNA‐Mediated Knockdown, and Lentiviral Transduction

The femurs and tibiae were isolated from 8‐week‐old C57BL/6J mice. The bone marrow was aspirated, and cells were resuspended in MACS buffer, filtered through a 30 μm strainer, and enriched for CD11b‐positive cells using magnetic‐activated cell sorting (MACS). Bone marrow‐derived macrophages (BMMs) were cultured in complete DMEM supplemented with 10% fetal bovine serum (FBS) and 1% penicillin–streptomycin. After 3 days, RANKL (50 ng/mL) and M‐CSF (30 ng/mL) were added to induce osteoclast formation. The RAW264.1 cell line was cultured in DMEM with 10% FBS and 1% penicillin–streptomycin. When the cells reached approximately 80% confluence, they were dissociated with 0.25% trypsin and resuspended in fresh medium. RANKL (50 ng/mL) and M‐CSF (30 ng/mL) were added to differentiate OCs, and the cells were cultured for 7 days to generate mature OCs.

ITGB2‐ and Rac1‐specific siRNAs were purchased from HanBio (Shanghai, China). siNC was used as a negative control. Cells were transfected with siRNA using Lipofectamine 3000 (Cat. No. 31985062; Thermo Fisher Scientific) according to the manufacturer's instructions and incubated for 48 h for subsequent experimental treatments of BMM and RAW264.7. The specific sequences are listed in Table S1.

Lentiviral vectors (pEZ‐Lv193) from GeneCopoeia were used to overexpress ITGB1, with the OmicsLinkTM Expression Clone (EX‐Mm03355‐Lv193) and Negative Clone (EX‐NEG‐Lv193). Cells were co‐transfected using Lipofectamine 3000 according to the manufacturer's instructions. Subsequent experimental treatments of BMM and RAW264.7 cells were performed 48 h post‐transfection. Specific sequences and vector information are detailed in Table S2.

### 2.6 Co‐Immunoprecipitation (Co‐IP)

Interactions between RAC1 and ITGB2 and between ITGA2 and ITGB2/ITGB1 were detected using a Co‐IP kit (Cat. No. 88804, Thermo Fisher, USA). The BMMs were lysed with lysis buffer, and the supernatant was collected after centrifugation. Equal amounts of lysates were incubated with Protein A/G agarose beads conjugated to specific antibodies (ITGB2, RAC1, ITGA2, or ITGB1). A control with a non‐specific antibody was included to account for non‐specific binding. The precipitated complexes were washed, analysed using SDS‐PAGE, and transferred onto a membrane.

### 2.7 Bimolecular Fluorescence Complementation Assay (BiFC)

Bimolecular fluorescence complementation (BiFC) method was used to study the interactions between RAC1 and ITGB2 [22]. Expression vectors containing the N‐terminal (YFP‐N) and C‐terminal (YFP‐C) fragments of yellow fluorescent protein (YFP) were constructed by inserting RAC1 and ITGB2 at their ends. Plasmids were extracted from *E. coli* and their sequence accuracy was verified. The vectors were transfected into HEK293T cells and cultured for 48 h to achieve stable fluorescent protein expression. YFP fluorescence was observed in the transfected cells using confocal microscopy, indicating an RAC1‐ITGB2 interaction.

### 2.8 Rac1 Activity Assay

Rac1 activation was assessed using the Rac1‐GTP pull‐down kit (Cat. No. 16118, Thermo Fisher, USA). BMMs were lysed on ice with a lysis buffer containing a protease inhibitor, and the supernatants were collected after centrifugation. The protein concentration was quantified using a BCA protein assay kit. Equal amounts of lysates were incubated with GST‐tagged PAK1‐PBD magnetic beads at 4°C for 60 min. Bound Rac1‐GTP was eluted with elution buffer and analysed using western blotting (WB).

### 2.9 Western Blotting Analysis

After protein extraction, equal amounts of protein were separated by SDS‐PAGE and transferred onto polyvinylidene fluoride (PVDF) membranes. The membrane was blocked with a 5% skim milk or BSA solution to prevent non‐specific binding, then incubated overnight at 4°C with specific primary antibodies (see Table S3). After washing, secondary antibodies conjugated with chemiluminescent labels were used to detect bound antibodies. Finally, membranes were imaged and quantified using a chemiluminescence imaging system.

### 2.10 Fluorescence Analysis of F‐Actin, TRAP, and Microtubules in BMMs

Fluorescence staining was performed to investigate the expression and distribution of F‐actin, TRAP (tartrate‐resistant acid phosphatase), and microtubules in BMMs. F‐actin was stained with phalloidin‐fluorescein isothiocyanate (CTCC‐JD006; PH Biotechnology, China) after fixing with 4% paraformaldehyde for 15 min. The cells were washed with PBS and incubated with phalloidin for 30 min, followed by nuclear counterstaining. TRAP activity was assessed using a TRAP staining kit (CTCC‐JD005; PH Biotechnology, China) by incubating fixed cells with TRAP solution for 30–60 min, and TRAP‐positive cells were observed under a microscope. Microtubules were detected by fixing cells with 4% paraformaldehyde and incubating with α‐tubulin antibodies and fluorescence‐labelled secondary antibodies. Samples were viewed under a confocal microscope, and the distribution of F‐actin, TRAP‐positive cells, and microtubules was analysed using image analysis software.

### 2.11 Quantitative Real‐Time PCR

Total RNA was isolated from the cell lines using RNAiso Plus (Takara, 9109, China), followed by cDNA synthesis using the PrimeScript RT Reagent Kit (Takara, RR036Q, China). Quantitative real‐time PCR was performed using a SYBR kit (Q311‐02; Vazyme, China) in a 96‐well thermal cycler with a StepOne Plus real‐time PCR system (Bio‐Rad Laboratories). The primer details are provided in Table S4.

### 2.12 Micro‐CT for Bone Analysis

Micro‐CT imaging was performed using the SkyScan 1276 scanner (Bruker, Belgium S.A./N.V.) at a resolution of 9 μm, a voltage of 50 kV, a current of 500 μA, and a 1.0 mm aluminium filter. Data analysis was conducted using the CTAn version 1.9 software (Bruker, Belgium S.A./N.V.) to quantify parameters such as subchondral bone volume/total volume (B.V./T.V. %), and tissue mineral density (TMD). The region of interest was defined as a subsection of the subchondral bone's load‐bearing area, measuring 0.5 mm in medio‐lateral width and 1.0 mm in ventro‐dorsal length. Three‐dimensional reconstruction was performed using the CTVox version 3.3.1 software (Bruker, Belgium S.A./N.V.).

### 2.13 Statistical Analysis

Each experiment was repeated at least three times. Statistical analyses were conducted using the GraphPad Prism 8 software. Normality was assessed using the Shapiro–Wilk test. For comparisons between two groups, Student's *t*‐test (or Mann–Whitney *U* test for non‐normally distributed data) was used. For multiple comparisons, one‐way or two‐way analysis of variance (ANOVA) with Tukey's post hoc test was performed. Data are presented as mean ± SEM, with statistical significance set at *p* < 0.05.

## 3 Results

### 3.1 ITGB2 Exhibits a Negative Correlation With ITGB1 in Subchondral Bone

**FIGURE 1:**
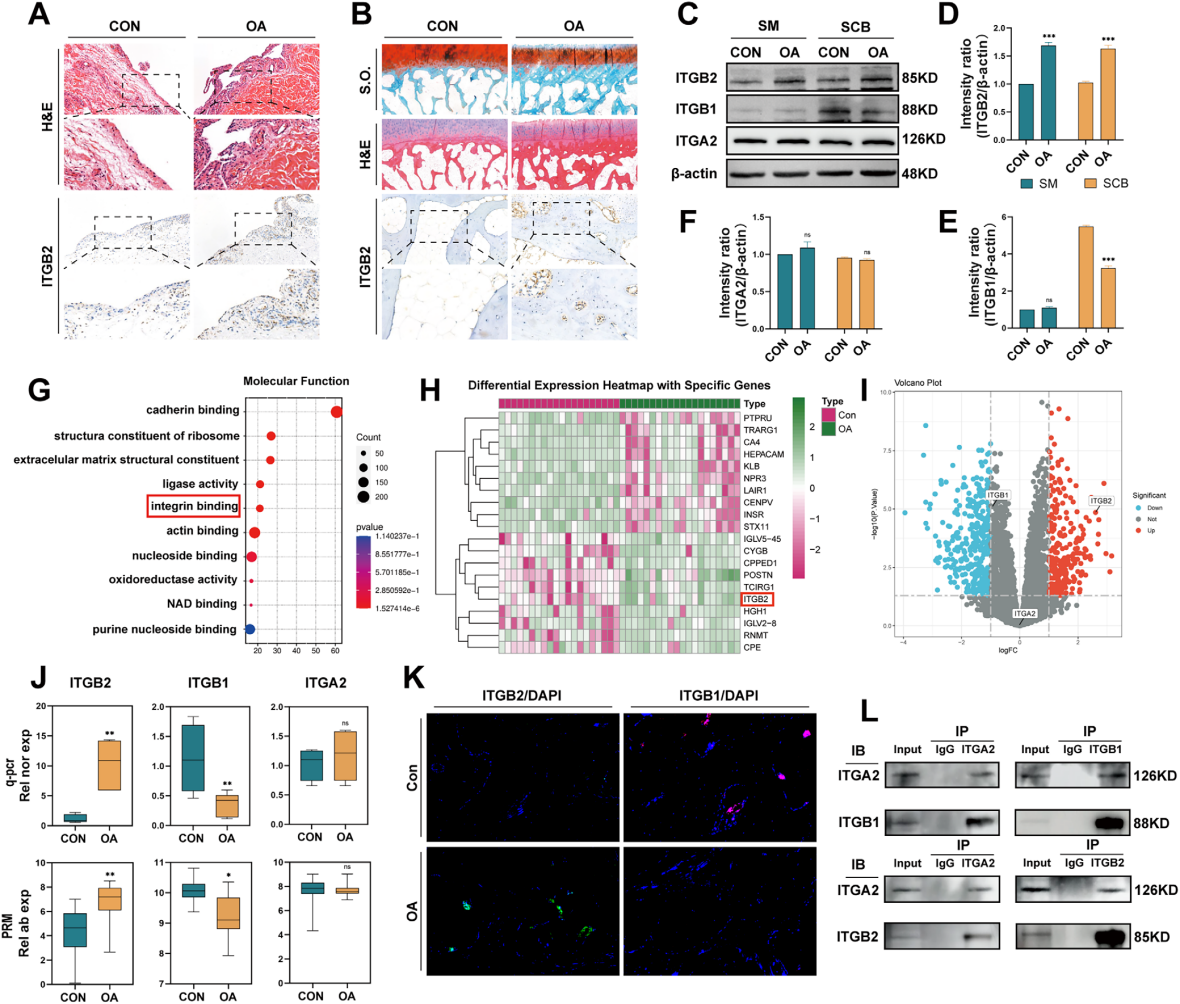
Negative correlation between ITGB2 and ITGB1 expression levels in the subchondral bone of the knee joint. (A) Haematoxylin and eosin (HE) staining of synovial tissues from healthy controls (CON, *n* = 3) and patients with TKA (OA, *n* = 3), and immunohistochemical (IHC) detection of ITGB2 protein expression. Scale bar: 200 μm. (B) Safranin O/Fast Green (SO/FG) staining of sagittal sections of non‐weight‐bearing (CON, *n* = 3) and weight‐bearing (OA, *n* = 3) subchondral tibial bone from patients with TKA, highlighting proteoglycans (red) and bone tissue (green). IHC analysis of ITGB2 expression in both groups. Scale bar: 200 μm. (C) Western blot analysis of protein expression in the subchondral bone and synovial tissues from CON (*n* = 3) and patients with OA (*n* = 3). (D–F) Quantitative analysis of ITGB2, ITGA2, and ITGB1 expression in the subchondral bone and synovial tissues from CON and patients with OA, compared with that of the control group. (G) Gene Ontology (GO) enrichment analysis of candidate hub genes. (H and I) Heatmap and volcano plot of differentially expressed proteins obtained through proteomic analysis, illustrating protein expression differences between OA and CON groups. (J) qRT‐PCR analysis of RNA isolated from the subchondral bone tissue of CON (*n* = 4) and patients with OA (*n* = 5). Box plot showing relative normalised expression values against GAPDH, calculated by the ΔCt method and compared with the control group. And box plot of target protein expression differences in the subchondral bone tissue samples from OA (*n* = 20) and CON (*n* = 20) groups based on PRM proteomics, comparing relative abundance (Rel ab exp) of the samples. (K) Immunostaining of ITGB1 or ITGB2 in the subchondral bone tissue from and CON (*n* = 3) patients with OA (*n* = 3) using specific antibodies, observed under fluorescence microscopy. DAPI was used for nuclear staining. Scale bar: 500 μm. (L) Co‐IP was performed in BMMs using ITGA2 antibody (or IgG) for positive immunoprecipitation, followed by reverse immunoprecipitation with ITGB1 antibody. The interaction between ITGA2 and ITGB1 was detected by Western blot. The same method was used for positive and reverse Co‐IP with ITGA2 and ITGB2 antibodies. *Error bars represent the mean ± standard deviation; statistical significance: ***p < 0.001, **p < 0.01, *p* < 0.05, ns, no significant difference. Comparisons between two groups were conducted using Student's *t*‐test.

To assess the correlation between ITGB2 and OA, we conducted a transcriptomic analysis using a public database (Figure S1). These results indicated that ITGB2 was significantly upregulated in OA synovial and subchondral bone tissues. These findings were validated in the human knee synovial and subchondral bone tissues. OA synovial and subchondral bone tissues exhibited higher ITGB2 expression than that of the controls (Figure 1A,B). Previous studies have reported that ITGA2, ITGB2, and ITGB1 are enriched in the human knee subchondral and synovial tissues [11, 23], indicating a potential interaction among these integrin subunits. To investigate this, we conducted protein interaction analysis using the STRING database. We identified 10 high‐confidence (combinatorial score ≥ 0.9; 2 nodes) interaction partners of human ITGA2, including ITGB2 (score = 0.96) and ITGB1 (score = 0.99) (Figure S2A). To confirm the interaction between ITGA2, ITGB2, and ITGB1, we performed co‐IP assays using total protein extracts from BMMs (Figure 1L). These results indicate that ITGA2 precipitated into ITGB2 and ITGB6. This validated our computational analysis and confirmed the interactions between these integrin subunits. To further investigate the correlation between the three integrin subunits, we performed a regression analysis on the synovial membrane and subchondral bone datasets from the GEO public database. The results indicated a negative correlation between ITGB2 and ITGB1 expression in the subchondral bone (Figure S2B, *R* ^2^ = 0.16, *p* < 0.00094). To verify this, we extracted proteins from synovial and subchondral bone tissues and analysed them using WB. The results demonstrated a negative correlation between ITGB2 and ITGB1 expression in the OA subchondral bone, with increased ITGB2 expression accompanied by reduced ITGB1 expression (Figure 1C–F). To clarify the correlation between integrins and OA, we performed a proteomic analysis of the human tibial subchondral bone. GO enrichment analysis identified integrin binding as a key function associated with differential gene expression (Figure 1G). The heatmap displays the top 20 genes, including ITGB2 (Figure 1H). The volcano plot showed that ITGB2 was upregulated as a differentially expressed protein in OA (*p* < 0.05, logFC > 1). ITGB1 was downregulated in patients with OA, but the difference was not statistically significant (*p* > 0.05, LogFC > 1). No significant differences in ITGA2 expression were observed (*p* > 0.05, logFC < 1) (Figure 1I). Subsequent qRT‐PCR, proteomics, and immunofluorescence staining of human subchondral bone tissue revealed that ITGB2 transcription and expression were enhanced in OA, whereas ITGB1 transcription and expression were decreased (Figure 1J,K). Based on these findings, we confirmed a negative correlation between ITGB2 and ITGB1 expression in the subchondral bone.

### 3.2 Direct Interaction of ITGB2 With Rac1 During Osteoclast Differentiation

**FIGURE 2:**
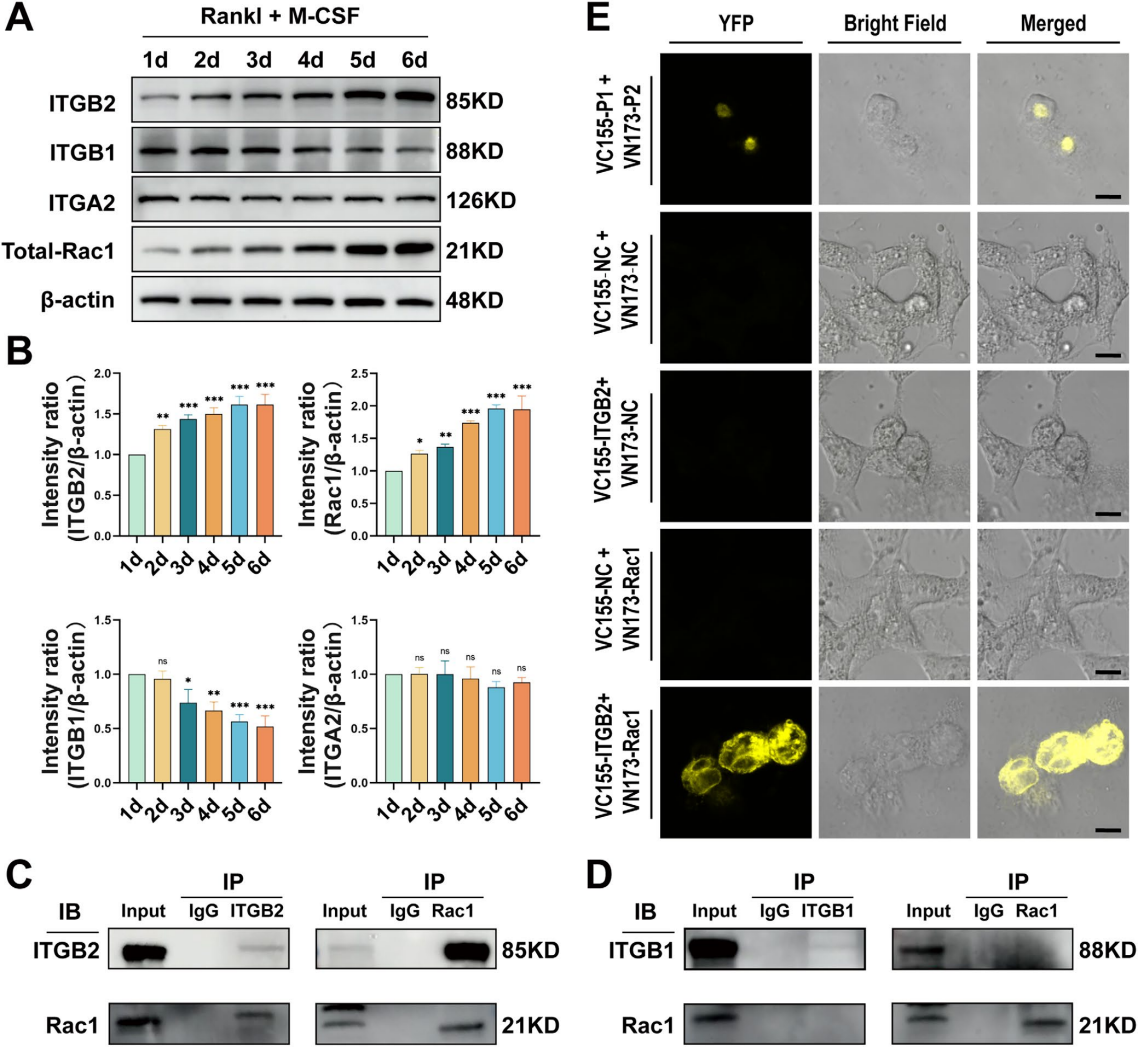
ITGB2 and Rac1 Co‐immunoprecipitation assay. (A) Bone marrow macrophages (BMMs) were treated with RANKL and M‐CSF at different time points, and the protein expression levels of the total protein extracts during osteoclastogenesis were detected by western blotting (*n* = 3). (B) Quantitative analysis of ITGB2, ITGB1, ITGA2, and Total‐Rac1 expression during osteoclastogenesis compared with the that of the 1d group. (C) After 24 h of osteoclast differentiation induction, positive Co‐IP was performed using an ITGB2 antibody (or IgG), followed by reverse immunoprecipitation with a Rac1 antibody. The interaction between ITGB2 and Rac1 was detected using western blotting. (D) The same method was applied using ITGB1 and Rac1 antibodies for positive and reverse co‐immunoprecipitation experiments, and the interaction between ITGB2 and Rac1 was detected using western blotting. (E) Co‐immunoprecipitation experiments (ITGB2‐Rac1) verified using the BiFC system. After co‐transfection of HEK293T cells with the reporter constructs under different conditions, antibody staining was performed and bioluminescence signals were observed. Scale bar: 10 μm. *Error bars represent the mean ± standard deviation; statistical significance: ***p < 0.001, **p < 0.01, *p* < 0.05, ns, no significant difference. Comparisons between two groups were conducted using Student's *t*‐test.

We further investigated whether ITGB2 and ITGB1 interact directly with Rac1 during osteoclast differentiation. We first established an osteoclast differentiation model. The results indicated that under RANKL and M‐CSF stimulation, BMMs differentiated into multinucleated OCs by day 5. By day 7, OCs were further fused and enlarged. The expression levels of osteoclast differentiation markers (NFATC1 and MMP9) also increased during the induction period (Figure S3). Next, to observe the expression of the three integrin subunits and Rac1 during osteoclast differentiation, we performed WB analysis of total proteins from BMMs 1–6 days after RANKL and M‐CSF stimulation. The results showed a time‐dependent increase in the expression of ITGB2 and Total‐Rac1 with osteoclastic differentiation, whereas ITGB1 expression remained unchanged in the presence of ITGA2 (Figure 2A,B). These results validated the negative correlation between ITGB2 and ITGA2 at the cellular and tissue level (Figure 1C–F,I–K). Previous studies have shown that the integrin receptor subunits that directly interact with Rac1 vary across different cellular contexts [24, 25]. To investigate whether Rac1 forms a complex with ITGB2 or ITGB1 during BMM differentiation into osteoclasts, we performed Co‐IP assays to detect Rac1 interactions with ITGB2 and ITGB1, and found that Rac1 co‐precipitated with ITGB2 (Figure 2C). In contrast, the ITGB1 antibody failed to co‐precipitate significant levels of Rac1 from BMM extracts that had initiated differentiation (Figure 2D). BiFC experiments were conducted to confirm the interaction between ITGB2 and Rac1. As expected, we obtained results consistent with those of the co‐IP experiments because ITGB2 interacted with Rac1 (Figure 2E). These results suggest that ITGB2 directly interacts with Rac1 during osteoclast differentiation, whereas ITGB1 does not participate in this process.

### 3.3 ITGB2 Regulates Osteoclast Differentiation via Rac1 Activation and Cross‐Talks With ITGB1

**FIGURE 3:**
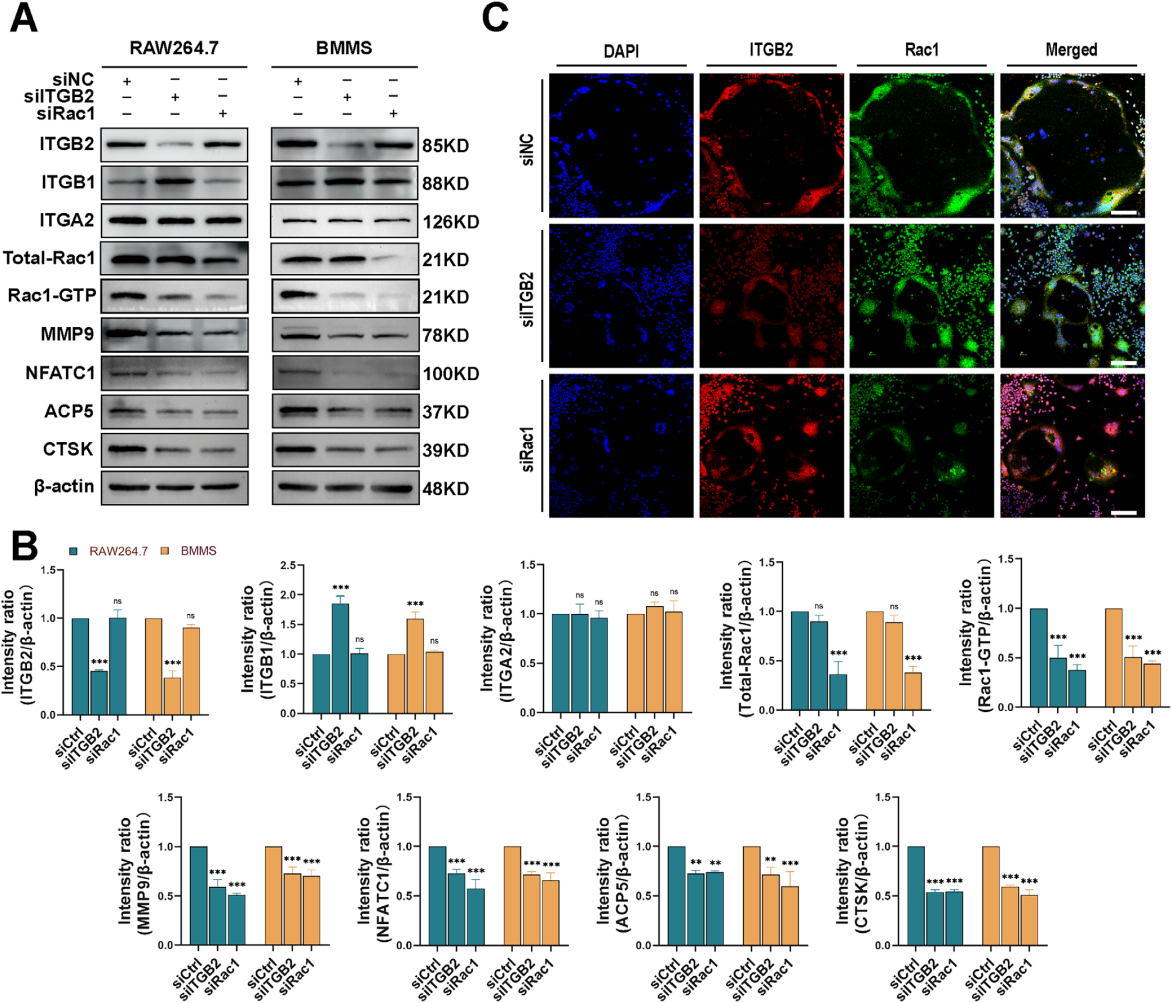
ITGB2 Deficiency Impairs RAC1 Activation and Inhibits Osteoclast Differentiation. (A) Bone marrow macrophages (BMMs) and RAW264.7 cells were treated under different conditions (siNC, siITGB2, and siRac1), followed by 7 days of induction with RANKL and M‐CSF. The protein expression levels of the total protein extracts during osteoclastogenesis were analysed using western blotting (*n* = 3). (B) Quantitative analysis of ITGB2, ITGB1, ITGA2, Total‐Rac1, Rac‐ATP, MMP9, NFATC1, ACP5, and CTSK expression during osteoclastogenesis compared to that of the control group. (C) Laser confocal microscopy images showing the expression of ITGB2 and Rac1 in BMMs treated with siITGB2 and siRac1 after 7 days of induction with RANKL and M‐CSF. Scale bar: 500 μm. Fluorescent labeling: Green represents Rac1, red represents ITGB2, and DAPI was used for nuclear staining (*n* = 3). *Error bars represent the mean ± standard deviation; statistical significance: ***p < 0.001, **p < 0.01, *p* < 0.05, ns, no significant difference. Comparisons between two groups were conducted using Student's *t*‐test.

To investigate the effects of ITGB2 and Rac1 on osteoclast differentiation, we used siRNAs (NC, ITGB2, and Rac1) to silence the gene expression in BMMs and RAW264.7 cells. To detect Rac1 activation, we first extracted total cellular protein and isolated the activated form of Rac1 (Rac1‐GTP) using a Rac1‐GTP pull‐down assay. Protein extracts from BMMs and RAW264.7 cells were analysed by WB, revealing a specific siRNA‐mediated lossoffunction of ITGB2, which reduced activated Rac1 (Rac1‐GTP) levels without affecting total Rac1 expression. ITGB2 or Rac1 knockdown decreases osteoclast levels of MMP9, NFATc1, ACP5, and CTSK (Figure 3A,B). These results suggested that ITGB2 regulates osteoclast differentiation by modulating Rac1 activation. Furthermore, immunofluorescence co‐staining of ITGB2 and Rac1 revealed that they colocalised to the membranes of differentiated osteoclasts (Figure 3C). Treatment with ITGB2 and Rac1 siRNAs reduced the membrane expression of both ITGB2 and Rac1, and F‐actin staining results indicated that ITGB2 and Rac1 siRNA treatment decreased osteoclast size and area, inhibiting osteoclastogenesis (Figure S4). These results suggested that ITGB2 modulates osteoclast differentiation by regulating Rac1 activation. Additionally, ITGB2 knockdown increased ITGB1 expression (Figure 3A,B). To further investigate the interplay between ITGB1 and ITGB2, we overexpressed ITGB1 in ITGB2‐knockdown osteoclast precursors. Immunofluorescence analysis revealed that this not only further suppressed ITGB2 expression but also reduced the levels of osteoclast differentiation markers (MMP9, NFATc1), indicating a potentiated inhibition of osteoclastogenesis (Figure S5).

### 3.4 ITGB2 Modulates Osteoclast Function by Regulating Microtubule Distribution and Actin Ring Formation

**FIGURE 4:**
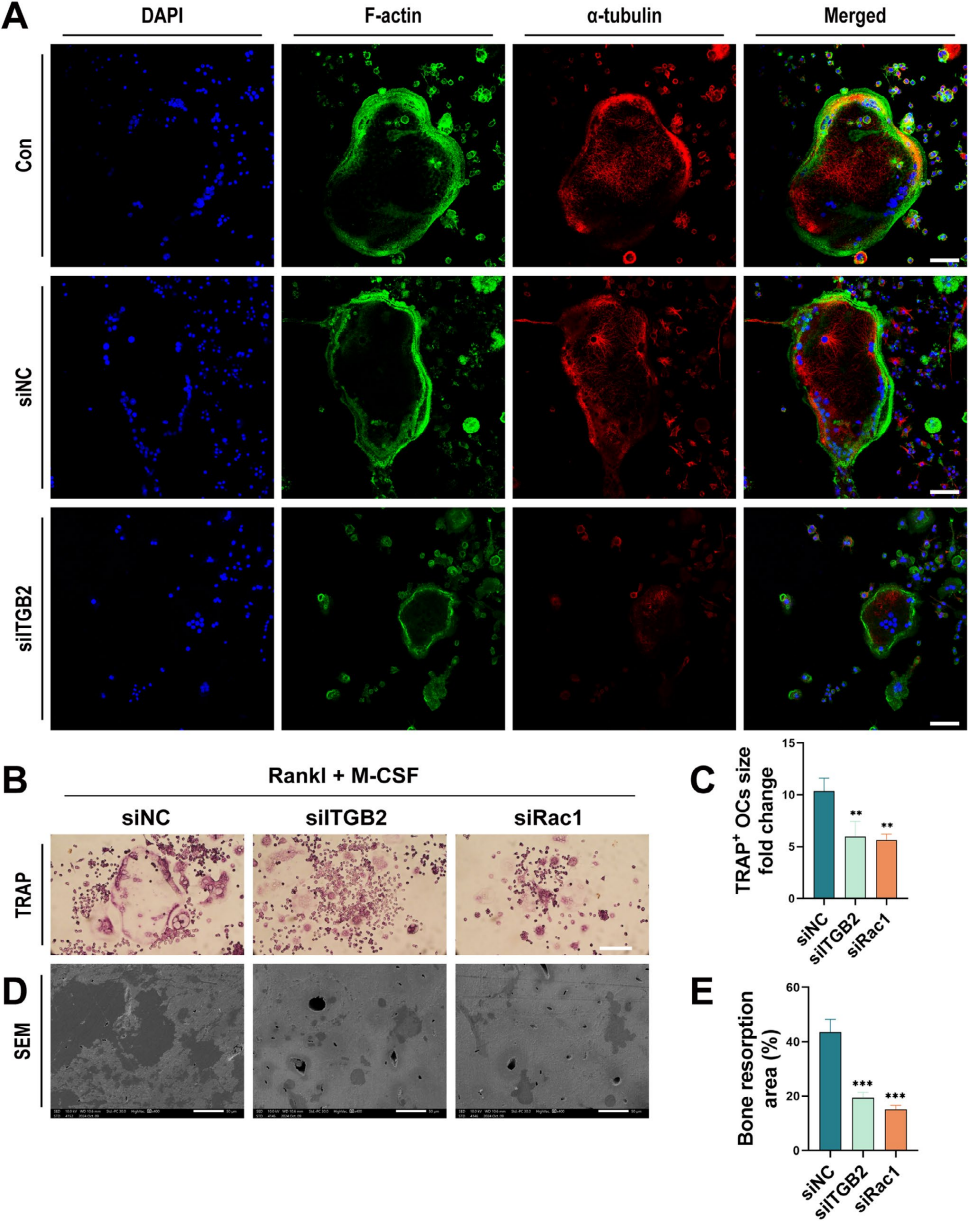
ITGB2 Deficiency affects Actin ring formation and microtubule migration to the cell periphery, leading to osteoclast dysfunction. (A) Laser confocal microscopy images showing the expression of F‐Actin and α‐tubulin in Bone marrow macrophages (BMMs) treated with siITGB2 after 7 days of induction with RANKL and M‐CSF. Fluorescent labeling: Green represents F‐Actin, red represents α‐tubulin, and DAPI was used for nuclear staining (*n* = 3). (B) BMMs were transfected with siNC (negative control), siITGB2, or siRac1, followed by 7 days of induction with RANKL and M‐CSF. TRAP staining revealed osteoclast formation (*n* = 3). Scale bar: 500 μm. (C) Quantitative analysis of the size of TRAP‐positive osteoclasts in different treatment groups (siNC, siITGB2, and siRac1) compared to the control group. (D) Scanning electron microscopy (SEM) was used to observe the bone resorption pits formed by osteoclasts on bone slices, showing osteoclast functionality (*n* = 3). Scale bar: 50 μm. (E) Quantitative analysis of the area of bone resorption pits in different treatment groups (siNC, siITGB2, and siRac1) compared to the control group.*Error bars represent the mean ± standard deviation; statistical significance: ***p < 0.001, **p < 0.01, *p* < 0.05, ns, no significant difference. Comparisons between two groups were conducted using Student's *t*‐test.

We further investigated whether siRNA‐mediated knockdown of ITGB2 affects actin ring formation and α‐tubulin distribution. siRNA (NC, ITGB2) was used to inhibit gene expression in BMMs, and cytoskeletal changes in osteoclasts were observed through F‐actin and α‐tubulin immunofluorescence staining. The results showed that F‐actin fibre network formation in osteoclasts was significantly inhibited in the siITGB2‐treated group. Meanwhile, α‐tubulin aggregated at the cell edges in the siITGB2 group compared to the Con and siNC groups. The siITGB2‐treated group showed altered α‐tubulin distribution, with less aggregation at the cell edges (Figure 4A). These findings suggest that ITGB2 is essential in osteoclast formation and function by regulating actin and microtubule remodelling during osteoclast differentiation.

ITGB's role on F‐actin and bone resorption assays was assessed by TRAP staining, F‐actin staining, and bone resorption. TRAP staining revealed that OCs in the siITGB2‐treated group were significantly smaller than those in the control and siNC groups (Figure 4B,C). F‐actin staining further confirmed that osteoclast differentiation in the siITGB2‐treated group was significantly reduced, as indicated by a smaller osteoclast boundary and diminished F‐actin rings, suggesting inhibition of osteoclast formation. Scanning electron microscopy revealed that the number and area of bone resorption pits were significantly lower in the siITGB2‐treated group than in the control and siNC groups (Figure 4D,E). These results indicate that ITGB2 is essential for osteoclast differentiation and function.

### 3.5 Upregulation of ITGB2 Is Associated With Increased Osteoclast Activity in Subchondral Bone of OA Mice

**FIGURE 5:**
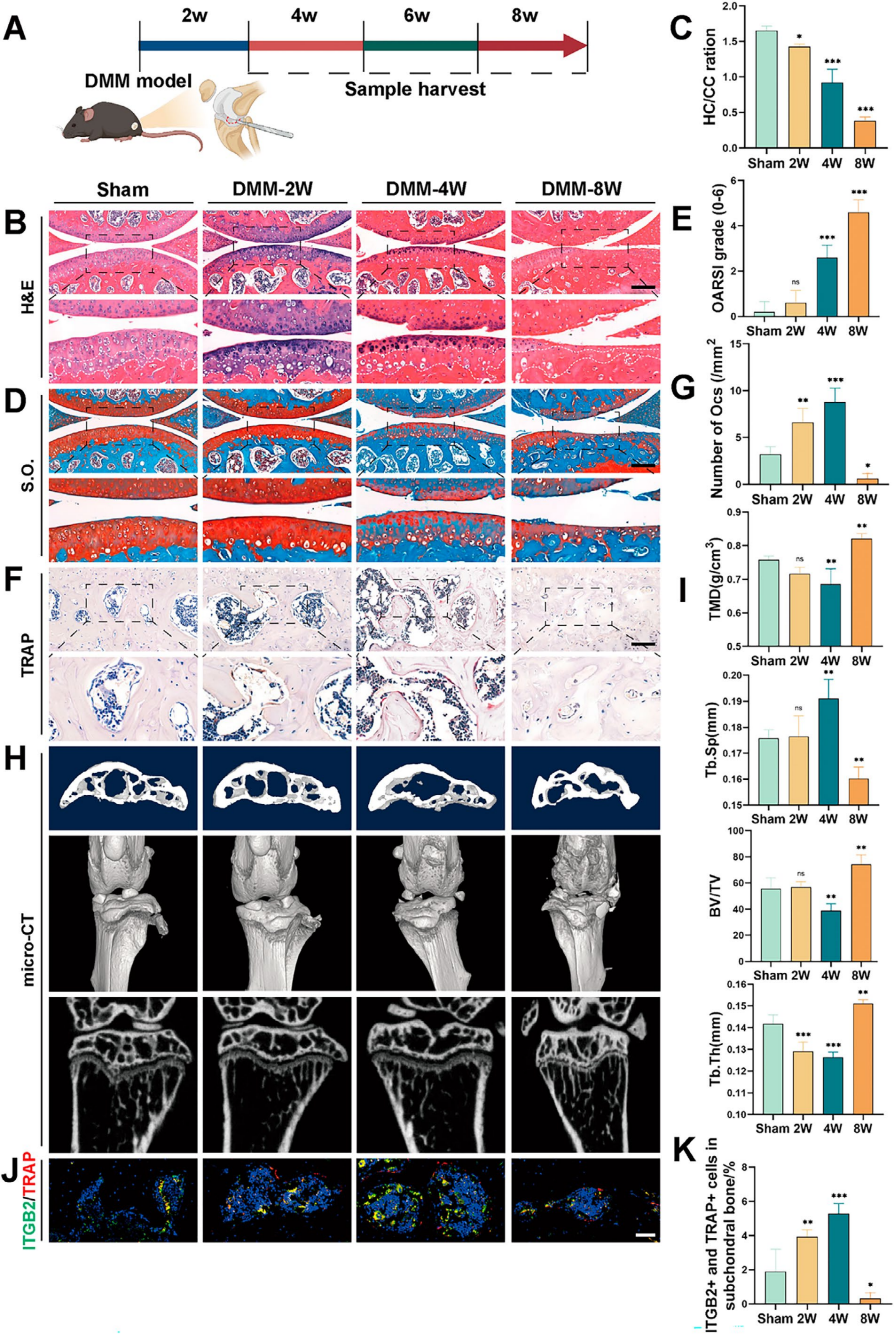
ITGB2 upregulation is associated with increased osteoclast activity in the subchondral bones of mice with Osteoarthritis. (A) Schematic representation of the DMM surgical procedure in wild type (WT) mice. (B) Haematoxylin and eosin (H&E) staining showing the morphological features of coronal sections of subchondral bone in tibiae from mice (*n* = 5). Scale bar: 200 μm. (C) Quantitative analysis of tibial cartilage thickness compared with the Sham group. (D) Safranin‐O/Fast Green staining of coronal sections of the subchondral bone in the tibiae of mice at different time points after DMM surgery (*n* = 5). Scale bar: 200 μm. (E) Quantitative analysis of OARSI scores for the tibial cartilage compared to the Sham group. (F) Representative TRAP‐stained images (*n* = 5). Scale bar: 20 μm. (G) Quantitative analysis of TRAP‐positive osteoclast density in the bone tissue compared to the Sham group. (H and I) 2D μ‐CT images and derived 3D and 2D reconstructed images of the subchondral bone structure at different time points after DMM surgery (*n* = 5). (K) Quantitative analysis of bone mineral density (TMD), trabecular thickness (Tb.Th), and subchondral bone mass (BV/TV) in the joint cartilage compared with the control group. (J) Immunofluorescent staining of ITGB2 and TRAP in the subchondral bone at different time points after DMM surgery (*n* = 5). Scale bar: 20 μm. (K) Quantitative analysis of the intensity of ITGB2 and TRAP double‐positive cells compared with the Sham group. *Error bars represent the mean ± standard deviation; statistical significance: ***p < 0.001, **p < 0.01, *p* < 0.05, ns, no significant difference. Comparisons between two groups were conducted using Student's *t*‐test.

To investigate the relationship between ITGB2 expression and osteoclast activity, we conducted observations at various time points in a mouse model of OA following surgically induced medial meniscus instability (DMM) (Figure 5A). H&E staining, Safranin O staining, and OARSI score analyses revealed mild articular cartilage damage at 2 weeks and worsening of surface damage at 4 and 8 weeks. At 8 weeks, the damage was further exacerbated, as indicated by a decrease in cartilage thickness and in the ratio of hyaline to calcified cartilage (Figure 5B–E). Additionally, TRAP staining showed that the number of TRAP‐positive OCs peaked at 4 weeks before declining (Figure 5F,G). Micro‐CT scans of the subchondral bone in DMM mice confirmed this observation, showing a significant decrease in bone mineral density (TMD) and bone volume fraction (BV/TV) along with an increase in trabecular separation (Tb.Sp) at 4 weeks. At week 8, there was a significant increase in the TMD and BV/TV, along with a decrease in Tb.Sp, accompanied by osteoclastogenesis (Figure 5H,I). Immunofluorescence analysis showed that the number of ITGB2/TRAP double‐positive OCs in the subchondral cartilage peaked at 4 weeks after DMM and declined at 8 weeks (Figure 5J,K). These results suggest that ITGB2 expression is closely linked to time‐dependent changes in osteoclast activity in the osteoarthritis mouse model.

### 3.6 ITGB2 Deficiency Mitigates the Progression of Osteoarthritis Induced by DMM in a Mouse Model

**FIGURE 6:**
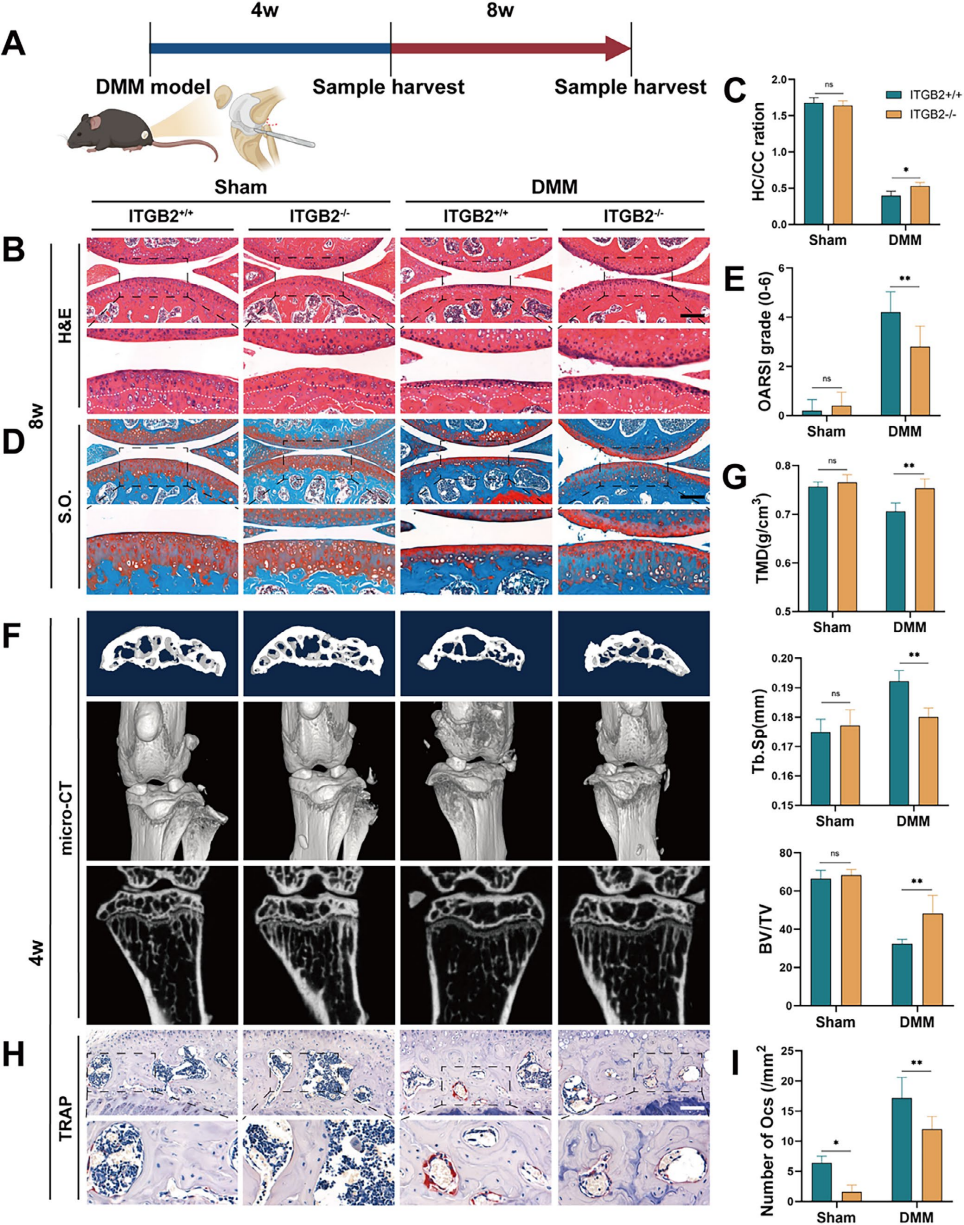
ITGB2 deficiency alleviates DMM‐induced osteoarthritis progression in mice. (A) Schematic of the experimental design for DMM surgery in KO mice. (B) Haematoxylin and eosin (H&E) staining of coronal sections of the subchondral bone in the tibiae 8 weeks after DMM surgery (*n* = 5). (C) Quantitative analysis of tibial cartilage thickness. Scale bar: 200 μm. (D) Safranin O/fast green staining of the subchondral bone in the tibia 8 weeks after DMM surgery (*n* = 5). Scale bar: 200 μm. (E) Quantitative analysis of OARSI scores for the tibial cartilage. (F) 2D μ‐CT images and derived 3D and 2D reconstructed images of subchondral bone at 4 weeks after DMM surgery, showing structural changes (*n* = 5). (G) Quantitative measurement of bone mineral density (TMD), trabecular thickness (Tb.Th), and subchondral bone mass (BV/TV) in the joint trabecular bone. (H) Representative images of TRAP staining 4 weeks after DMM surgery (*n* = 5). Scale bar: 20 μm. (I) Quantitative analysis of TRAP‐positive osteoclast density in the bone tissue. *Error bars represent the mean ± standard deviation; statistical significance: ***p < 0.001, **p < 0.01, *p* < 0.05, ns, no significant difference. Comparisons between two groups were conducted using Student's *t*‐test.

Next, we investigated the role of ITGB2 in OA pathogenesis using ITGB2 knockout mice. To assess the effect of ITGB2 knockout on overall health, we collected tissue samples from the heart, liver, spleen, lungs, and kidneys of adult ITGB2 knockout (KO) and wild‐type (WT) mice (3 months old) for comparison (Figure S6). Haematoxylin and eosin (H&E) staining revealed that under normal dietary conditions, the organ structures in ITGB2 knockout mice were not significantly different from those in WT mice. We performed DMM surgery in 8‐week‐old WT and KO mice (Figure 6A). H&E and Safranin‐O/Fast Green staining and OARSI score analysis showed similar articular cartilage conditions in both groups 8 weeks after sham surgery, with no significant degeneration observed. However, in the DMM‐induced OA model, cartilage degeneration was more severe in WT mice than in ITGB2 knockout mice. After 8 weeks, WT mice showed more pronounced hyaline cartilage degeneration, calcified cartilage hyperplasia, and higher OARSI scores (Figure 6B–E). Four weeks after the sham surgery, micro‐CT analysis of the mouse knees revealed no significant differences between the two groups in terms of TMD, Tb.Sp, or BV/TV of the subchondral bone. However, four weeks after DMM surgery, micro‐CT analysis showed that, compared to WT mice, ITGB2 knockout mice had significantly higher subchondral TMD and BV/TV, along with significantly lower Tb.Sp (Figure 6F,G). Moreover, the number of TRAP‐positive OCs in the subchondral bone of ITGB2 knockout mice was significantly lower than that in WT mice (Figure 6H,I). Taken together, these results suggest that ITGB2 deletion in BMMs significantly alleviates OA progression by inhibiting osteoclast activity.

## 4 Discussion

**FIGURE 7:**
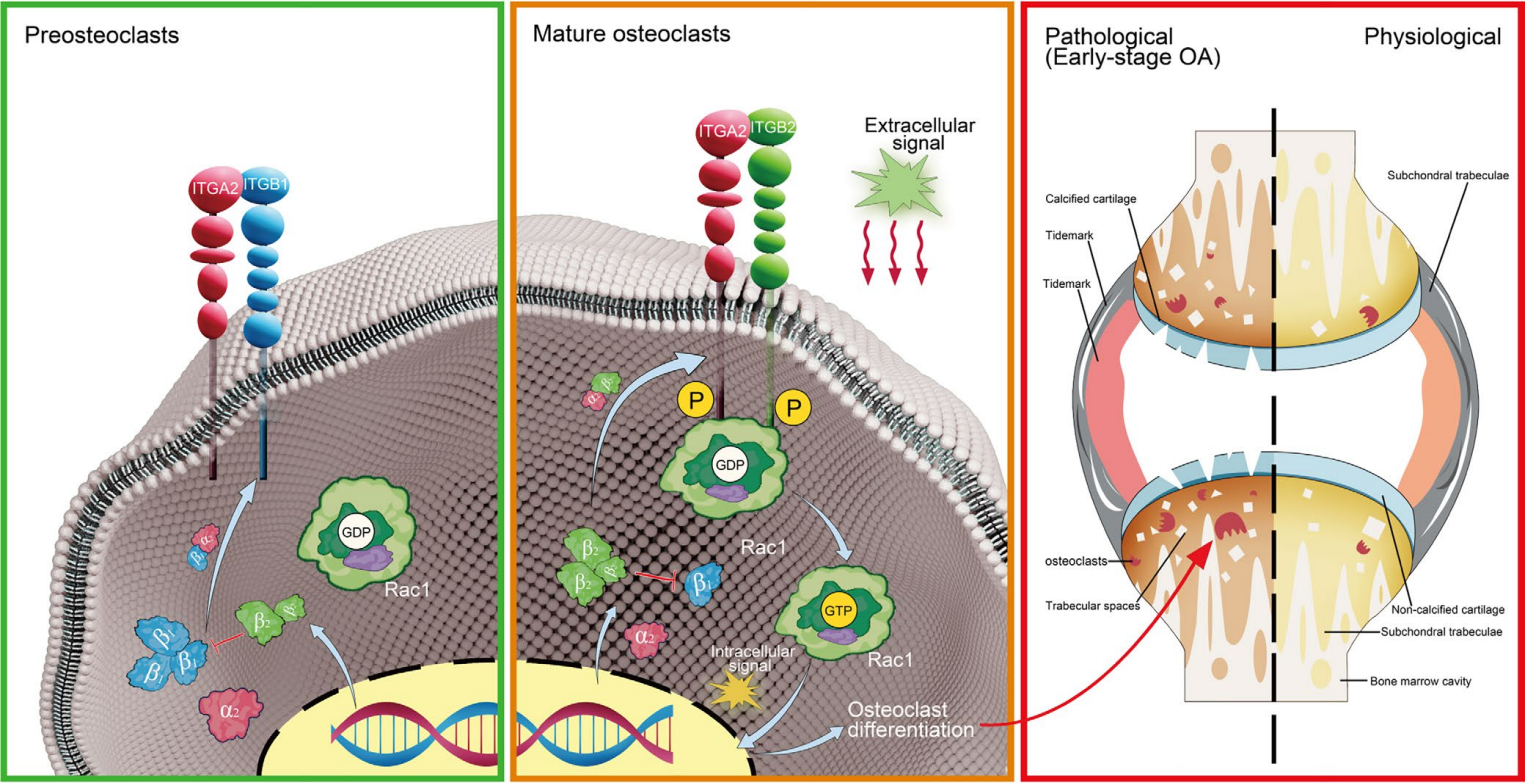
Schematic representation of the potential mechanism of ITGB2 in osteoarthritis (OA) development. Left panel: In osteoclast precursor cells, the transcription and translation levels of ITGB1 are higher than those of ITGB2, resulting in increased expression of ITGA2‐ITGB1 integrin receptors on the cell membrane. Middle panel: In OA, extracellular signalling stimuli increased the transcription and translation levels of ITGB2 while inhibiting the transcription and translation of ITGB1. This leads to an increased expression of ITGA2‐ITGB1 integrin receptors on the membrane. Subsequently, Rac1 is activated by binding to the tail of ITGA2‐ITGB1 and by signals transduced from integrin receptors, converting it into Rac‐GTP. Rac‐GTP induces differentiation of osteoclasts into mature ones Right panel: Excessive activation of osteoclasts in the subchondral bone promotes OA progression.

The pathological mechanisms underlying OA are complex and diverse. Recent studies have indicated that the enhanced differentiation of OCs in the subchondral bone is a key factor in the development and progression of OA, suggesting that targeting osteoclast differentiation in subchondral bone is a promising strategy for OA treatment. Our transcriptomic and proteomic analyses of human subchondral bone revealed that ITGB2 expression was significantly increased in OA and was negatively correlated with ITGB1 expression. During osteoclast differentiation, ITGB2 interacts with and activates Rac1, and inhibition of this process suppresses osteoclast differentiation. Additionally, we identified an integrin inter‐regulatory mechanism by which ITGB2 negatively regulates ITGB1 levels during osteoclast differentiation. In a mouse model, ITGB2 expression positively correlated with osteoclastogenesis. ITGB2 knockdown inhibits osteoclastogenesis and reduces osteochondral bone abnormal remodelling in the subchondral bone, thereby slowing OA progression (Figure 7).

The expression of the ITGB2‐encoding gene is significantly upregulated in OA and correlates with osteoclast function, as reported in several transcriptomic studies [26, 27, 28]. These results are consistent with our findings. In this study, we analysed human OA subchondral bone samples using multi‐omics approaches and found that both the transcriptional and translational levels of ITGB2 were significantly increased in patients with OA compared to controls. β2 integrins are heterodimeric surface receptors typically formed by the combination of ITGB2 with one of four different α‐subunits (ITGAM, ITGAL, ITGAD, and ITGAX). The ITGB2‐ITGAM complex is highly expressed in monocytes. However, our study revealed that ITGA2 and ITGB2 combine to form functional complexes in BMMs. This finding is consistent with a previous study by Karla et al., who reported that ITGB2 and ITGA2 specifically bind and are highly expressed in SCLC [29]. Moreover, our comprehensive review of integrin‐related studies revealed that complexes formed by homologous ITGA with different ITGB subunits or by different ITGA with the same ITGB can have conflicting effects on OA and related cartilage and bone metabolism. For instance, activating ITGA1‐ITGB1 receptors or blocking ITGA2‐ITGB1 integrins can reduce the pathology scores and catabolic factors in OA cartilage, suggesting functional competition among specific integrin subunits. Additionally, stimulation with ITGAV‐ITGB5 or inhibition of ITGAV‐ITGB3 effectively suppresses osteoclast differentiation [10, 11]. These results indicated functional competition among specific integrin subunits during OA progression. We observed contrasting expression patterns of ITGB2 and ITGB1 during osteoclast differentiation and in the OA subchondral bone. Specifically, ITGB2 knockdown in OCs resulted in increased levels of ITGB1. This implies competition between ITGA2‐ITGB2 and ITGA2‐ITGB1 during osteoclast differentiation. Therefore, it would be valuable to investigate whether the switch from ITGA2‐ITGB1 to ITGA2‐ITGB2 in OA affects the differentiation of OC precursors into mature OCs. However, previous studies indicate that blockade of the ITGA2‐ITGB1 integrin inhibits IL‐7‐induced inflammatory bone loss and reduces osteoclast numbers by decreasing Th17 cell numbers in the bone marrow and lowering the production of IL‐17 and NF‐κB ligand receptor activators [14]. Considering the fact that both ITGA2‐ITGB2 and ITGA2‐ITGB1 functionally promote osteoclast differentiation, it is possible that competition between ITGA2‐ITGB2 and ITGA2‐ITGB1 may be nonfunctional. The transition between these two states may arise from a shift between the inflammatory and non‐inflammatory environments.

Numerous studies have indicated that the function of a subset of integrins within a single cell can be regulated by a mechanism termed integrin crosstalk, involving another integrin subset. For instance, adhesion molecules with dual IG structural domains (DICAM) preferentially bind to ITGB3, inhibiting the formation of the ITGAV‐ITGB3 dimer and thus obstructing osteoclastogenesis in downstream signalling pathways [30]. Although we observed a correlation between competitive integrin regulation and osteoclast differentiation as well as OA phenotypes, we ruled out the possibility of integrin crosstalk. Our observations indicated that during OCs differentiation, the expression of ITGA2 remained constant, whereas ITGB1 and ITGB2 expression levels exhibited significant changes, with ITGB1 increasing and ITGB2 decreasing. This suggests that the issue lies not in the competitive binding of cytokines to ITGB2 or ITGB1 but rather that certain endogenous factors likely regulate the transcription and expression of these integrin subunit genes, providing varying opportunities for competitive binding with different ITGA subunits. Previous studies demonstrated that specific transcription factors such as Snail and Slug regulate the expression of various integrin subunits within the same cell [31]. This underscores the fact that integrin regulation is influenced by both external factors and a complex network of endogenous signalling pathways. Future research should explore whether similar mechanisms are involved in the transition from ITGA2‐ITGB1 to ITGA2‐ITGB2 during the histological or cellular changes in OA.

The cytoplasmic domain of integrin ITGB (β‐tails) is essential as a link between integrins and Rac1 signalling pathways [32]. Additionally, some ITGB β‐tails connect to Rac1 through complexes such as ILK/paxillin/p95PKL/PIX and ILK/PINCH/Nck2/Dock180, activating it [33, 34, 35]. Therefore, we deduce that different ITGB β‐tails may have specific affinities for binding with RAC1. Our experimental results support this perspective, indicating that ITGB2, but not ITGB1, interacts with RAC1 during osteoclast differentiation, thereby influencing osteoclastogenesis. Additionally, Rac1 activity increased in an ITGB2‐dependent manner. Miao et al. [32] observed a similar phenomenon in CHO cells, in which ITGB1, rather than ITGB3, activated Rac1 upon adhesion to fibronectin. This suggests that in specific cell types or environmental conditions, ITGB2 preferentially interacts with RAC1, whereas ITGB1 may engage in other signalling pathways.

The bone resorption function of OCs relies on the integrity of the actin cytoskeleton and the formation of actin‐rich sealing zones [36]. Many studies have demonstrated that ITGAV‐ITGB3 mediates osteoclast adhesion to bone matrix proteins and regulates cytoskeletal reorganisation [37, 38, 39]. Other integrins, including ITGAV‐ITGB5 and ITGA9‐ITGB1, are present in osteoclast precursors and regulate their formation and function [40]. Rac1 regulates cytoskeletal reorganisation by binding to integrins [16]. These findings strongly indicate that ITGB2 and Rac1 influence osteoclast differentiation. This was confirmed by our findings that inhibiting ITGB2 or Rac1 attenuates osteoclast differentiation and reduces bone resorption. Microtubules are primarily composed of α‐ and β‐tubulin proteins, and their dynamics significantly affect cell morphology and function. During osteoclast differentiation, microtubules migrate from the cell centre to the periphery, facilitating shape changes that optimise their binding to the bone matrix. Furthermore, the migration of microtubules toward the cell edge directs actin organisation and polymerisation, forming a robust cytoskeletal support structure. This process is critical for osteoclast function and mobility [41]. Rac1 is closely linked to microtubule stability and plays a key role in cell migration and polarisation by enhancing the interaction between microtubules and actin rings [42, 43]. In our study, inhibition of ITGB2 reduced GTP‐RAC1 expression and impeded the migration of α‐tubulin toward the cell edge, as well as the formation of F‐actin. This may be one of the mechanisms by which ITGB2 influences osteoclast differentiation and bone resorption. However, our understanding of the role of microtubules in osteoclast activation and function remains limited. Inhibition of microtubule protein acetylation impairs the formation and stability of podosome belts and actin rings in OCs, thereby affecting bone resorption [44]. Further studies are necessary to determine whether ITGB2 influences OCs by regulating microtubule function.

The pathogenesis of OA is complex; however, its onset and progression are closely linked to cellular communication and cell‐matrix interactions. Increasing evidence indicates that interactions involving signalling molecules, integrins, and their ligands promote mechanotransduction and are vital for subchondral bone metabolism [45]. Although the relationship between ITGB1 and ITGB3 dysfunction and OA pathogenesis has been extensively reported [46, 47], the significance of ITGB2 in OA pathogenesis remains unclear. Current research on ITGB2 in OA reveals that under pathological conditions, ITGB2 expression levels increase in the cartilage, synovium, and subchondral bone tissue [20]. Studies have suggested that ITGB2 regulates immune and inflammatory responses by inhibiting the TLR signalling pathway. In the absence of ITGB2, TLR stimulation significantly increases the secretion of IL‐12 and IL‐6 in macrophages and dendritic cells, indicating that ITGB2 is crucial for regulating TLR‐mediated inflammatory responses [48]. Activation of the TLR/NF‐κB signalling pathway is known to increase osteoclastogenesis and activity, thereby promoting bone resorption. Concurrently, pro‐inflammatory factors in the inflammatory environment inhibit OB function, disrupting the balance between OBs and OCs and resulting in the loss and alteration of the subchondral bone [49]. These results suggest that ITGB2 contributes not only to cell adhesion but also to the regulation of signalling pathways involved in OA development. In this study, the DMM mouse model was used as a crucial platform. DMM surgery simulates functional dysfunction of the knee joint by introducing asymmetric mechanical loading, leading to a series of pathological changes [50]. Our study confirmed that knocking out ITGB2 significantly inhibited osteoclastogenesis and positively affected the progression of OA. Quantitative analysis using micro‐CT and histological staining demonstrated that ITGB2 knockout impacts osteoclastogenesis and delays OA progression in both normal physiological conditions and the DMM‐induced OA mouse model. In contrast, while ITGB2 knockout alleviates OA in the DMM model, it does not alter the OA phenotype in mice under normal physiological conditions. This paradox suggests that, under normal physiological conditions, the body may suppress excessive osteoclast activation by maintaining low ITGB2 expression in the subchondral bone, thus preventing early OA progression. Thus, studying ITGB2 not only provides new insights into OA mechanisms but also underscores the significance of targeting specific integrin subunits in OA.

In conclusion, our findings indicated that targeting ITGB2 may effectively slow the progression of OA. Furthermore, we identified the mechanisms through which integrins regulate osteoclastic differentiation and interact with each other in the subchondral bone of patients with OA. Despite the importance of these findings, this study has some limitations. First, we investigated the interaction between ITGB2 and Rac1, and future studies should further examine the role of ITGB in OA subchondral bone and its interactions with other signalling pathways. Moreover, although we demonstrated reciprocal regulation between ITGB2 and ITGB1, further studies are required to elucidate the specific mechanisms underlying this regulation.

## Author Contributions

Qunhua Jin, Xue Lin and Yang Yang conceived the study and designed the experiments. Yang Yang, Rui Sun, Zhibin Lan, Gang Wu and Qi Ma performed the experiments, acquired the data and analysed the results. Ye Ma, Xiaolei Chen, Jiangbo Yan, Long Ma, Xiaoxin He, Xiaoyi Ma and Kuanmin Tian provided technical consultation and contributed to data interpretation. Yang Yang and Rui Sun prepared the article. Qunhua Jin, Di Xue, Zhirong Chen and Yajing Su revised the manuscript and Supporting Information in the process of revision. Yang Yang and Rui Sun contributed equally to this work.

## Ethics Statement

The study protocol was approved by the Medical Research Ethics Review Committee of the General Hospital of Ningxia Medical University. Informed consent was obtained from all participants prior to their inclusion in the study. All animal experiments were conducted in accordance with the requirements and approvals of the Animal Ethics and Welfare Committee of the Experimental Animal Center of Ningxia Medical University.

## Conflicts of Interest

The authors declare no conflicts of interest.

## Supporting information


**Figure S1:** Identification of key genes involved in osteoarthritis (OA). (A) Heatmap and volcano plot of differentially expressed genes (DEGs) in the GSE1919 (upper) and GSE55235 (lower) datasets (green points represent downregulated genes, grey points represent genes with no significant difference, and red points represent upregulated genes). (B) Weighted Gene Co‐expression Network Analysis (WGCNA) was performed on GSE1919 (left) and GSE55235 (right) datasets to identify OA‐related genes. The upper panel shows a hierarchical clustering dendrogram based on the Topological Overlap Matrix (1‐TOM) for all genes in both datasets. Each branch of the dendrogram represents a gene, and co‐expression modules are shown in different colours. The lower left panel shows the module‐trait heatmap, which displays the correlation between gene modules and OA with corresponding correlation coefficients and *p* values. The lower‐right panel shows a scatter plot of the turquoise module, which is common to both datasets and has the strongest positive correlation with OA. (C) Venn diagram showing 76 overlapping candidate hub genes. (D) The 10 most significantly upregulated genes obtained from the PPI network. (E) Nomogram model of hub genes and ROC curve (F) were used to evaluate the diagnostic performance of our nomogram and each hub gene in the external dataset GSE51588.

**Figure S2:** Human ITGA2 interactome is also associated with osteoarthritis (OA). (A) Computer simulation analysis of human ITGA2 interactors using STRING 10.0. Red boxes indicate ITGA2, ITGB2, and ITGB1. Line colour represents known (turquoise), predicted (green), gene fusion (red), gene co‐occurrence (blue), or experimental (purple) interactions. KEGG pathway enrichment analysis, including that of the ITGA2 interactome, revealed significant enrichment in the ECM‐receptor interaction pathway. (B) Linear regression analysis of the normalised expression in human subchondral bone tissues (GSE51588 dataset) and synovial tissues (GSE55235 dataset) from the knee joints, examining the correlation between ITGA2, ITGB2, and ITGB1.

**Figure S3:** Establishment of osteoclast differentiation model. (A) Bone marrow macrophages (BMMs) were induced with RANKL and M‐CSF for different periods. TRAP staining was used to visualise osteoclast formation and laser confocal microscopy was used to observe the formation of actin rings in osteoclasts (*n* = 3). Scale bar: 100 μm. (B) Quantitative analysis of size changes in TRAP‐positive osteoclasts and the area of actin ring formation in osteoclasts. (C) Western blotting analysis of MMP9 and NFATC1 protein expression in BMMs at different time points after induction (*n* = 3). (D) Quantitative analysis of ITGB2, ITGB1, ITGA2, and Total‐Rac1 expression during osteoclastogenesis compared with the 1d group. *Error bars represent the mean ± standard deviation; statistical significance: ***p < 0.001, **p < 0.01, *p* < 0.05, ns, no significant difference. Comparisons between two groups were conducted using Student's *t*‐test.

**Figure S4:** ITGB2 and Rac1 deficiency inhibits actin ring formation and leads to osteoclast differentiation defects. (A) BMMs and RAW264.7 cells were treated under different conditions (siNC, siITGB2, and siRac1), followed by 7 days of induction with RANKL and M‐CSF. Immunofluorescence staining was performed to assess the effects of these treatments on osteoclast differentiation. The upper panel shows DAPI staining of the nuclei (blue), the middle panel shows F‐actin distribution (green), and the bottom panel shows a merged image of the nuclei and cytoskeleton. Scale bar: 100 μm.

**Figure S5:** Overexpression of ITGB1 further reduces ITGB2 expression and potentiates the inhibitory effect of ITGB2 deficiency on osteoclast differentiation. (A) Bone marrow‐derived macrophages (BMMs) were treated under different conditions (siNC, siITGB2, and siITGB2 + OE‐ITGB1), followed by induction with RANKL and M‐CSF for 7 days. Immunofluorescence staining was performed to evaluate the effects of these treatments on osteoclast differentiation and protein expression during differentiation. Nuclei were stained with DAPI (blue), and protein distributions are shown in red. Scale bar: 100 μm. OE, overexpression. Quantitative analysis of fluorescence intensities for (B) ITGB2, (C) ITGB1, (D) MMP9, and (E) NFATc1 proteins. *Error bars represent the mean ± standard deviation; statistical significance: ***p < 0.001, **p < 0.01, *p* < 0.05, ns, no significant difference. Comparisons between two groups were conducted using Student's *t*‐test.

**Figure S6:** Effect of ITGB2 Knockout on the heart, liver, spleen, lungs, and kidney structure. (A) Haematoxylin and eosin (H&E) staining was performed on the heart, liver, spleen, lungs, and kidneys before DMM surgery to assess histological features. The results showed no significant changes in the basic structural characteristics compared to those of the sham surgery group.

**Table S1:** Real‐time PCR primers.

**Table S2:** Details of Itgb1 gene sequence and control, linked to pEZ—Lv193 vector.

**Table S3:** Summary of antibodies employed in the study.

**Table S4:** Gene identification primers.

## Data Availability

The data that support the findings of this study are available from the corresponding author upon reasonable request.

## References

[1] Y. Yang , Z. Lan , J. Yan , et al., “Effect of Intra‐Knee Injection of Autologous Adipose Stem Cells or Mesenchymal Vascular Components on Short‐Term Outcomes in Patients With Knee Osteoarthritis: An Updated Meta‐Analysis of Randomized Controlled Trials,” Arthritis Research and Therapy 25 (2023): 147. 37563715 10.1186/s13075-023-03134-3 PMC10413774

[2] W. H. Robinson , C. M. Lepus , Q. Wang , et al., “Low‐Grade Inflammation as a Key Mediator of the Pathogenesis of Osteoarthritis,” Nature Reviews Rheumatology 12 (2016): 580–592. 27539668 10.1038/nrrheum.2016.136 PMC5500215

[3] V. P. Leifer , J. N. Katz , and E. Losina , “The Burden of OA‐Health Services and Economics,” Osteoarthritis and Cartilage 30 (2022): 10–16. 34023527 10.1016/j.joca.2021.05.007 PMC8605034

[4] M. Favero , E. Belluzzi , A. Ortolan , et al., “Erosive Hand Osteoarthritis: Latest Findings and Outlook,” Nature Reviews Rheumatology 18 (2022): 171–183. 35105980 10.1038/s41584-021-00747-3

[5] H. Madry , “The Subchondral Bone: A New Frontier in Articular Cartilage Repair,” Knee Surgery, Sports Traumatology, Arthroscopy 18 (2010): 417–418. 10.1007/s00167-010-1071-y 20127311

[6] L. J. Raggatt and N. C. Partridge , “Cellular and Molecular Mechanisms of Bone Remodeling,” Journal of Biological Chemistry 285 (2010): 25103–25108. 20501658 10.1074/jbc.R109.041087 PMC2919071

[7] A. Shibakawa , K. Yudoh , K. Masuko‐Hongo , T. Kato , K. Nishioka , and H. Nakamura , “The Role of Subchondral Bone Resorption Pits in Osteoarthritis: MMP Production by Cells Derived From Bone Marrow,” Osteoarthritis and Cartilage 13 (2005): 679–687. 15961327 10.1016/j.joca.2005.04.010

[8] G. Zhen and X. Cao , “Targeting TGFbeta Signaling in Subchondral Bone and Articular Cartilage Homeostasis,” Trends in Pharmacological Sciences 35 (2014): 227–236. 24745631 10.1016/j.tips.2014.03.005 PMC4058854

[9] H. Rao , G. Lu , H. Kajiya , et al., “Alpha9beta1: A Novel Osteoclast Integrin That Regulates Osteoclast Formation and Function,” Journal of Bone and Mineral Research 21 (2006): 1657–1665. 16995821 10.1359/JBMR.060718 PMC1937336

[10] H. Jin , S. Jiang , R. Wang , Y. Zhang , J. Dong , and Y. Li , “Mechanistic Insight Into the Roles of Integrins in Osteoarthritis,” Frontiers in Cell and Development Biology 9 (2021): 693484. 10.3389/fcell.2021.693484 PMC8250141 34222261

[11] L. Mao , L. Wang , J. Xu , and J. Zou , “The Role of Integrin Family in Bone Metabolism and Tumor Bone Metastasis,” Cell Death Discovery 9 (2023): 119. 37037822 10.1038/s41420-023-01417-x PMC10086008

[12] E. K. Song , J. Jeon , D. G. Jang , et al., “ITGBL1 Modulates Integrin Activity to Promote Cartilage Formation and Protect Against Arthritis,” Science Translational Medicine 10 (2018): 462. 10.1126/scitranslmed.aam7486 30305454

[13] C. L. Elsegood , Y. Zhuo , G. A. Wesolowski , J. A. Hamilton , G. A. Rodan , and L. T. Duong , “M‐CSF Induces the Stable Interaction of cFms With alphaVbeta3 Integrin in Osteoclasts,” International Journal of Biochemistry and Cell Biology 38 (2006): 1518–1529. 16600665 10.1016/j.biocel.2006.02.011

[14] A. M. El , C. Arseneault , M. Boisvert , et al., “Cooperation Between IL‐7 Receptor and Integrin alpha2beta1 (CD49b) Drives Th17‐Mediated Bone Loss,” Journal of Immunology 195 (2015): 4198–4209. 10.4049/jimmunol.1500437 26408663

[15] N. A. Hotchin and A. Hall , “Regulation of the Actin Cytoskeleton, Integrins and Cell Growth by the Rho Family of Small GTPases,” Cancer Surveys 27 (1996): 311–322. 8909807

[16] S. E. LaFlamme , S. Mathew‐Steiner , N. Singh , D. Colello‐Borges , and B. Nieves , “Integrin and Microtubule Crosstalk in the Regulation of Cellular Processes,” Cellular and Molecular Life Sciences 75 (2018): 4177–4185. 30206641 10.1007/s00018-018-2913-x PMC6182340

[17] M. Zhu , B. H. Sun , K. Saar , et al., “Deletion of Rac in Mature Osteoclasts Causes Osteopetrosis, an Age‐Dependent Change in Osteoclast Number, and a Reduced Number of Osteoblasts In Vivo,” Journal of Bone and Mineral Research 31 (2016): 864–873. 26496249 10.1002/jbmr.2733 PMC4826801

[18] H. Wang , T. Li , Y. Jiang , et al., “Force‐Loaded Cementocytes Regulate Osteoclastogenesis via S1P/S1PR1/Rac1 Axis,” Journal of Dental Research 102 (2023): 1376–1386. 37735908 10.1177/00220345231195765

[19] B. L. Walling and M. Kim , “LFA‐1 in T Cell Migration and Differentiation,” Frontiers in Immunology 9 (2018): 952. 29774029 10.3389/fimmu.2018.00952 PMC5943560

[20] T. Hu , Z. Zhang , C. Deng , X. Ma , and X. Liu , “Effects of beta2 Integrins on Osteoclasts, Macrophages, Chondrocytes, and Synovial Fibroblasts in Osteoarthritis,” Biomolecules 12 (2022): 1653. 36359003 10.3390/biom12111653 PMC9687144

[21] K. Tian , X. He , X. Lin , et al., “Unveiling the Role of Sik1 in Osteoblast Differentiation: Implications for Osteoarthritis,” Molecular and Cellular Biology 44 (2024): 411–428. 39169784 10.1080/10985549.2024.2385633 PMC11485870

[22] M. Chen , M. H. Wang , X. G. Shen , et al., “Neuropilin‐1 Facilitates Pseudorabies Virus Replication and Viral Glycoprotein B Promotes Its Degradation in a Furin‐Dependent Manner,” Journal of Virology 96 (2022): e131822. 10.1128/jvi.01318-22 PMC9599266 36173190

[23] Y. T. Konttinen , T. F. Li , J. W. Xu , et al., “Expression of Laminins and Their Integrin Receptors in Different Conditions of Synovial Membrane and Synovial Membrane‐Like Interface Tissue,” Annals of the Rheumatic Diseases 58 (1999): 683–690. 10531072 10.1136/ard.58.11.683 PMC1752798

[24] J. D. Humphries , A. Byron , M. D. Bass , et al., “Proteomic Analysis of Integrin‐Associated Complexes Identifies RCC2 as a Dual Regulator of Rac1 and Arf6,” Science Signaling 2 (2009): a51. 10.1126/scisignal.2000396 PMC2857963 19738201

[25] B. U. Sehgal , P. J. DeBiase , S. Matzno , et al., “Integrin Beta4 Regulates Migratory Behavior of Keratinocytes by Determining Laminin‐332 Organization,” Journal of Biological Chemistry 281 (2006): 35487–35498. 16973601 10.1074/jbc.M606317200 PMC2820731

[26] A. M. Selim , Y. A. Elsabagh , M. M. El‐Sawalhi , N. A. Ismail , and M. A. Senousy , “Association of Integrin‐Beta2 Polymorphism and Expression With the Risk of Rheumatoid Arthritis and Osteoarthritis in Egyptian Patients,” BMC Medical Genomics 16 (2023): 204. 37644537 10.1186/s12920-023-01635-3 PMC10463674

[27] W. Qian and Z. Li , “Expression and Diagnostic Significance of Integrin Beta‐2 in Synovial Fluid of Patients With Osteoarthritis,” Journal of Orthopaedic Surgery 31 (2023): 773419085. 10.1177/10225536221147213 37379363

[28] B. Hopwood , A. Tsykin , D. M. Findlay , and N. L. Fazzalari , “Microarray Gene Expression Profiling of Osteoarthritic Bone Suggests Altered Bone Remodelling, WNT and Transforming Growth Factor‐Beta/Bone Morphogenic Protein Signalling,” Arthritis Research and Therapy 9 (2007): R100. 17900349 10.1186/ar2301 PMC2212557

[29] K. Rubio , A. J. Romero‐Olmedo , P. Sarvari , et al., “Non‐Canonical Integrin Signaling Activates EGFR and RAS‐MAPK‐ERK Signaling in Small Cell Lung Cancer,” Theranostics 13 (2023): 2384–2407. 37215577 10.7150/thno.79493 PMC10196829

[30] Y. K. Jung , S. W. Han , G. W. Kim , J. H. Jeong , H. J. Kim , and J. Y. Choi , “DICAM Inhibits Osteoclast Differentiation Through Attenuation of the Integrin alphaVbeta3 Pathway,” Journal of Bone and Mineral Research 27 (2012): 2024–2034. 22492581 10.1002/jbmr.1632

[31] F. E. Turner , S. Broad , F. L. Khanim , et al., “Slug Regulates Integrin Expression and Cell Proliferation in Human Epidermal Keratinocytes,” Journal of Biological Chemistry 281 (2006): 21321–21331. 16707493 10.1074/jbc.M509731200

[32] H. Miao , S. Li , Y. L. Hu , et al., “Differential Regulation of Rho GTPases by Beta1 and Beta3 Integrins: The Role of an Extracellular Domain of Integrin in Intracellular Signaling,” Journal of Cell Science 115 (2002): 2199–2206. 11973360 10.1242/jcs.115.10.2199

[33] C. E. Turner , “Paxillin Interactions,” Journal of Cell Science 113 (2000): 4139–4140. 11069756 10.1242/jcs.113.23.4139

[34] C. Wu and S. Dedhar , “Integrin‐Linked Kinase (ILK) and Its Interactors: A New Paradigm for the Coupling of Extracellular Matrix to Actin Cytoskeleton and Signaling Complexes,” Journal of Cell Biology 155 (2001): 505–510. 11696562 10.1083/jcb.200108077 PMC2198863

[35] E. Kiyokawa , Y. Hashimoto , S. Kobayashi , H. Sugimura , T. Kurata , and M. Matsuda , “Activation of Rac1 by a Crk SH3‐Binding Protein, DOCK180,” Genes and Development 12 (1998): 3331–3336. 9808620 10.1101/gad.12.21.3331 PMC317231

[36] S. L. Teitelbaum , “The Osteoclast and Its Unique Cytoskeleton,” Annals of the New York Academy of Sciences 1240 (2011): 14–17. 22172034 10.1111/j.1749-6632.2011.06283.x

[37] S. I. Kim , Y. H. Kim , B. G. Kang , et al., “Linarin and Its Aglycone Acacetin Abrogate Actin Ring Formation and Focal Contact to Bone Matrix of Bone‐Resorbing Osteoclasts Through Inhibition of Alphavbeta3 Integrin and Core‐Linked CD44,” Phytomedicine 79 (2020): 153351. 32987362 10.1016/j.phymed.2020.153351

[38] M. Kim , J. Lin , J. E. Huh , et al., “Tetraspanin 7 Regulates Osteoclast Function Through Association With the RANK/alphavbeta3 Integrin Complex,” Journal of Cellular Physiology 237 (2022): 846–855. 34407208 10.1002/jcp.30559

[39] K. P. McHugh , K. Hodivala‐Dilke , M. H. Zheng , et al., “Mice Lacking Beta3 Integrins Are Osteosclerotic Because of Dysfunctional Osteoclasts,” Journal of Clinical Investigation 105 (2000): 433–440. 10683372 10.1172/JCI8905 PMC289172

[40] E. G. Estell , P. T. Le , Y. Vegting , et al., “Irisin Directly Stimulates Osteoclastogenesis and Bone Resorption In Vitro and In Vivo,” eLife 9 (2020): 9. 10.7554/eLife.58172 PMC7444909 32780016

[41] H. Liu , R. Zhang , S. Y. Ko , et al., “Microtubule Assembly Affects Bone Mass by Regulating Both Osteoblast and Osteoclast Functions: Stathmin Deficiency Produces an Osteopenic Phenotype in Mice,” Journal of Bone and Mineral Research 26 (2011): 2052–2067. 21557310 10.1002/jbmr.419

[42] K. J. Hamill , S. B. Hopkinson , P. DeBiase , and J. C. Jones , “BPAG1e Maintains Keratinocyte Polarity Through beta4 Integrin‐Mediated Modulation of Rac1 and Cofilin Activities,” Molecular Biology of the Cell 20 (2009): 2954–2962. 19403692 10.1091/mbc.E09-01-0051 PMC2695802

[43] Q. Jian , Y. Miao , L. Tang , et al., “Rab23 Promotes Squamous Cell Carcinoma Cell Migration and Invasion via Integrin beta1/Rac1 Pathway,” Oncotarget 7 (2016): 5342–5352. 26716504 10.18632/oncotarget.6701 PMC4868690

[44] O. Destaing , F. Saltel , B. Gilquin , et al., “A Novel Rho‐mDia2‐HDAC6 Pathway Controls Podosome Patterning Through Microtubule Acetylation in Osteoclasts,” Journal of Cell Science 118 (2005): 2901–2911. 15976449 10.1242/jcs.02425

[45] I. P. Geoghegan , D. A. Hoey , and L. M. McNamara , “Integrins in Osteocyte Biology and Mechanotransduction,” Current Osteoporosis Reports 17, no. 4 (2019): 195–206. 31250372 10.1007/s11914-019-00520-2

[46] H. Hendesi , M. F. Barbe , F. F. Safadi , M. A. Monroy , and S. N. Popoff , “Integrin Mediated Adhesion of Osteoblasts to Connective Tissue Growth Factor (CTGF/CCN2) Induces Cytoskeleton Reorganization and Cell Differentiation,” PLoS One 10 (2015): e115325. 10.1371/journal.pone.0115325 PMC4340870 25714841

[47] X. Lu , Y. Ito , P. Atsawasuwan , et al., “Ameloblastin Modulates Osteoclastogenesis Through the Integrin/ERK Pathway,” Bone 54 (2013): 157–168. 23385480 10.1016/j.bone.2013.01.041 PMC5023015

[48] N. K. Yee and J. A. Hamerman , “Beta(2) Integrins Inhibit TLR Responses by Regulating NF‐kappaB Pathway and p38 MAPK Activation,” European Journal of Immunology 43 (2013): 779–792. 23310953 10.1002/eji.201242550 PMC3809911

[49] G. Yang , K. Wang , H. Song , et al., “Celastrol Ameliorates Osteoarthritis via Regulating TLR2/NF‐kappaB Signaling Pathway,” Frontiers in Pharmacology 13 (2022): 963506. 36034791 10.3389/fphar.2022.963506 PMC9399520

[50] S. S. Glasson , T. J. Blanchet , and E. A. Morris , “The Surgical Destabilization of the Medial Meniscus (DMM) Model of Osteoarthritis in the 129/SvEv Mouse,” Osteoarthritis and Cartilage 15 (2007): 1061–1069. 17470400 10.1016/j.joca.2007.03.006

