# Correction to “Mechanism of ITGB2 in Osteoclast Differentiation in Osteoarthritis”

**DOI:** 10.1111/cpr.70229

**Published:** 2026-05-18

Y. Yang , R. Sun , Z. Lan , et al., “Mechanism of ITGB2 in Osteoclast Differentiation in Osteoarthritis,” Cell Proliferation 59, no. 3 (2026): e70107, 10.1111/cpr.70107.40730513 PMC12961538

In Section 3.4 of the Results, the second electron microscopy (EM) image in Figure 4D was incorrect due to duplication. This corrected EM image is reproduced below:

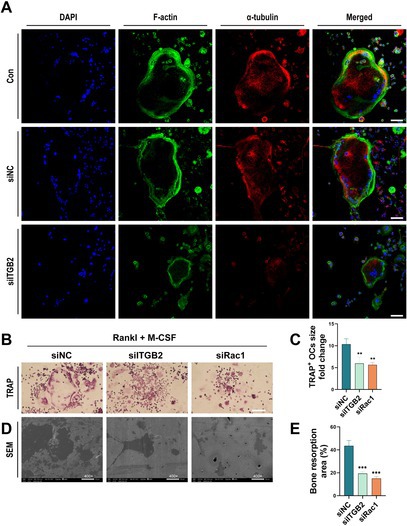

We apologize for this error.

