# Supplementary figures and images for: Mechanism of ITGB2 in Osteoclast Differentiation in Osteoarthritis
Source: Cell Prolif. 2025 Jul 29;59(3):e70107. doi: 10.1111/cpr.70107 (PMC12961538; doi:10.1111/cpr.70107)

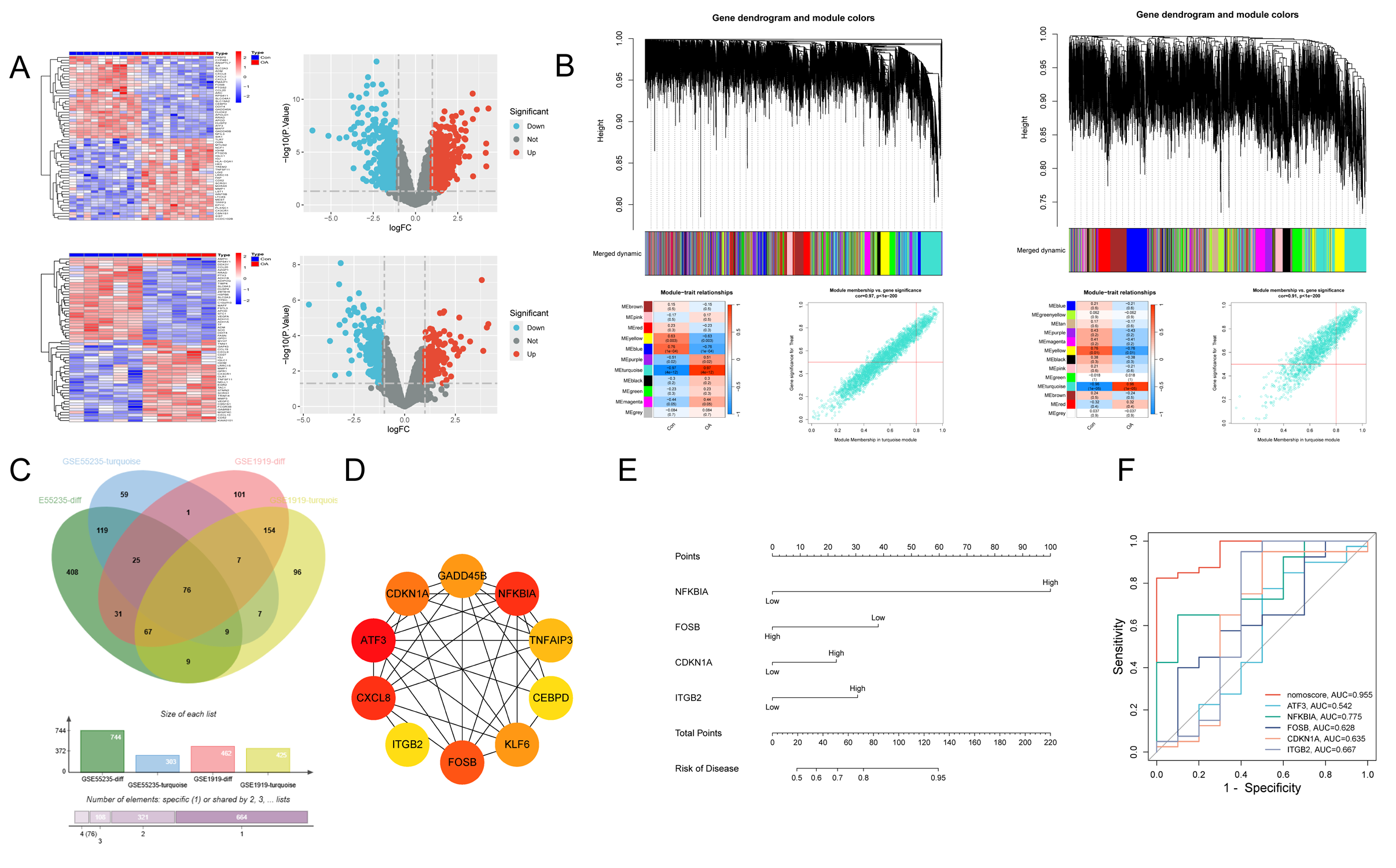

Supplement: Supplementary file 1 — Figure S1: Identification of key genes involved in osteoarthritis (OA). (A) Heatmap and volcano plot of differentially expressed genes (DEGs) in the GSE1919 (upper) and GSE55235 (lower) datasets (green points represent downregulated genes, grey points represent genes with no significant difference, and red points represent upregulated genes). (B) Weighted Gene Co‐expression Network Analysis (WGCNA) was performed on GSE1919 (left) and GSE55235 (right) datasets to identify OA‐related genes. The upper panel shows a hierarchical clustering dendrogram based on the Topological Overlap Matrix (1‐TOM) for all genes in both datasets. Each branch of the dendrogram represents a gene, and co‐expression modules are shown in different colours. The lower left panel shows the module‐trait heatmap, which displays the correlation between gene modules and OA with corresponding correlation coefficients and p values. The lower‐right panel shows a scatter plot of the turquoise module, which is common to both datasets and has the strongest positive correlation with OA. (C) Venn diagram showing 76 overlapping candidate hub genes. (D) The 10 most significantly upregulated genes obtained from the PPI network. (E) Nomogram model of hub genes and ROC curve (F) were used to evaluate the diagnostic performance of our nomogram and each hub gene in the external dataset GSE51588. [file CPR-59-e70107-s001.tif]

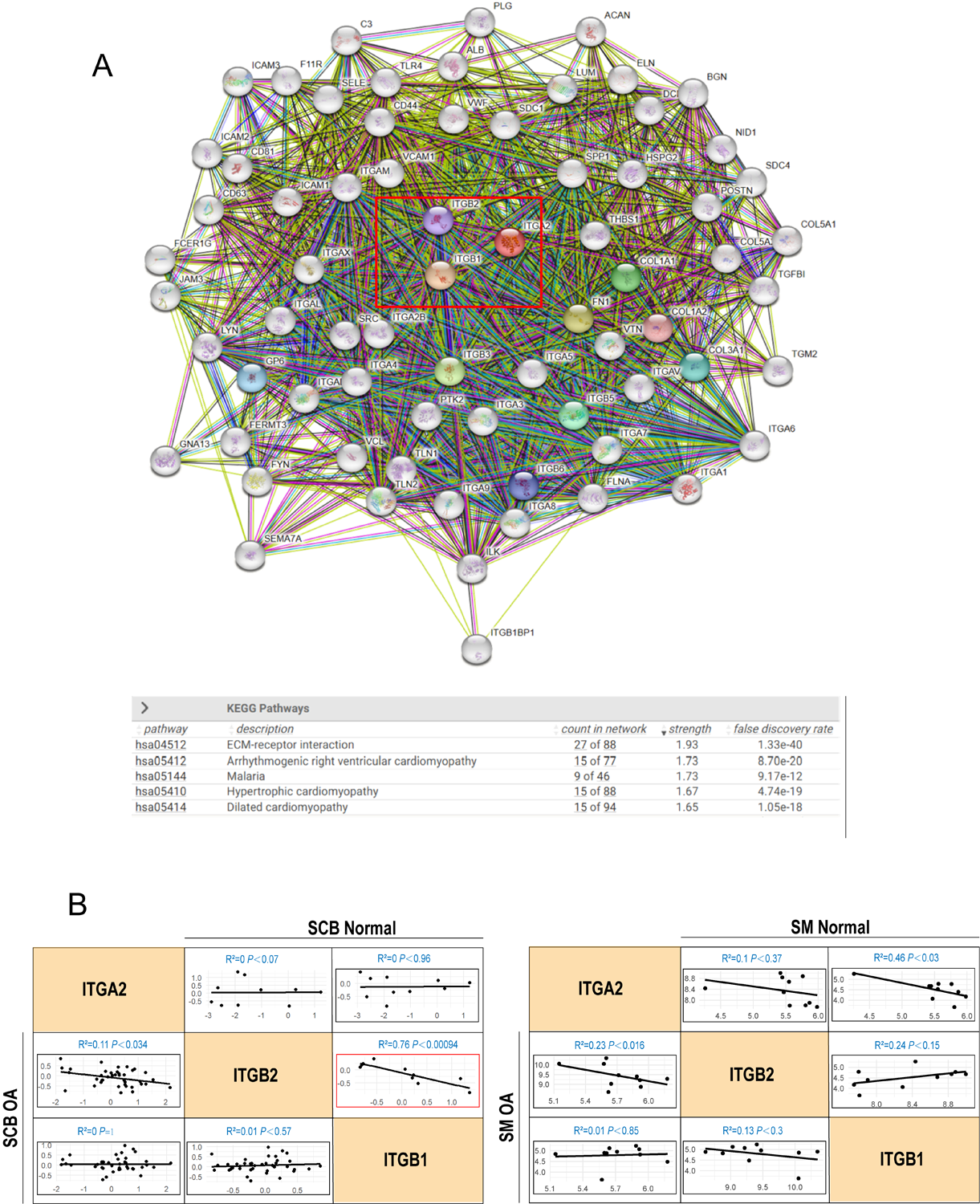

Supplement: Supplementary file 2 — Figure S2: Human ITGA2 interactome is also associated with osteoarthritis (OA). (A) Computer simulation analysis of human ITGA2 interactors using STRING 10.0. Red boxes indicate ITGA2, ITGB2, and ITGB1. Line colour represents known (turquoise), predicted (green), gene fusion (red), gene co‐occurrence (blue), or experimental (purple) interactions. KEGG pathway enrichment analysis, including that of the ITGA2 interactome, revealed significant enrichment in the ECM‐receptor interaction pathway. (B) Linear regression analysis of the normalised expression in human subchondral bone tissues (GSE51588 dataset) and synovial tissues (GSE55235 dataset) from the knee joints, examining the correlation between ITGA2, ITGB2, and ITGB1. [file CPR-59-e70107-s004.tif]

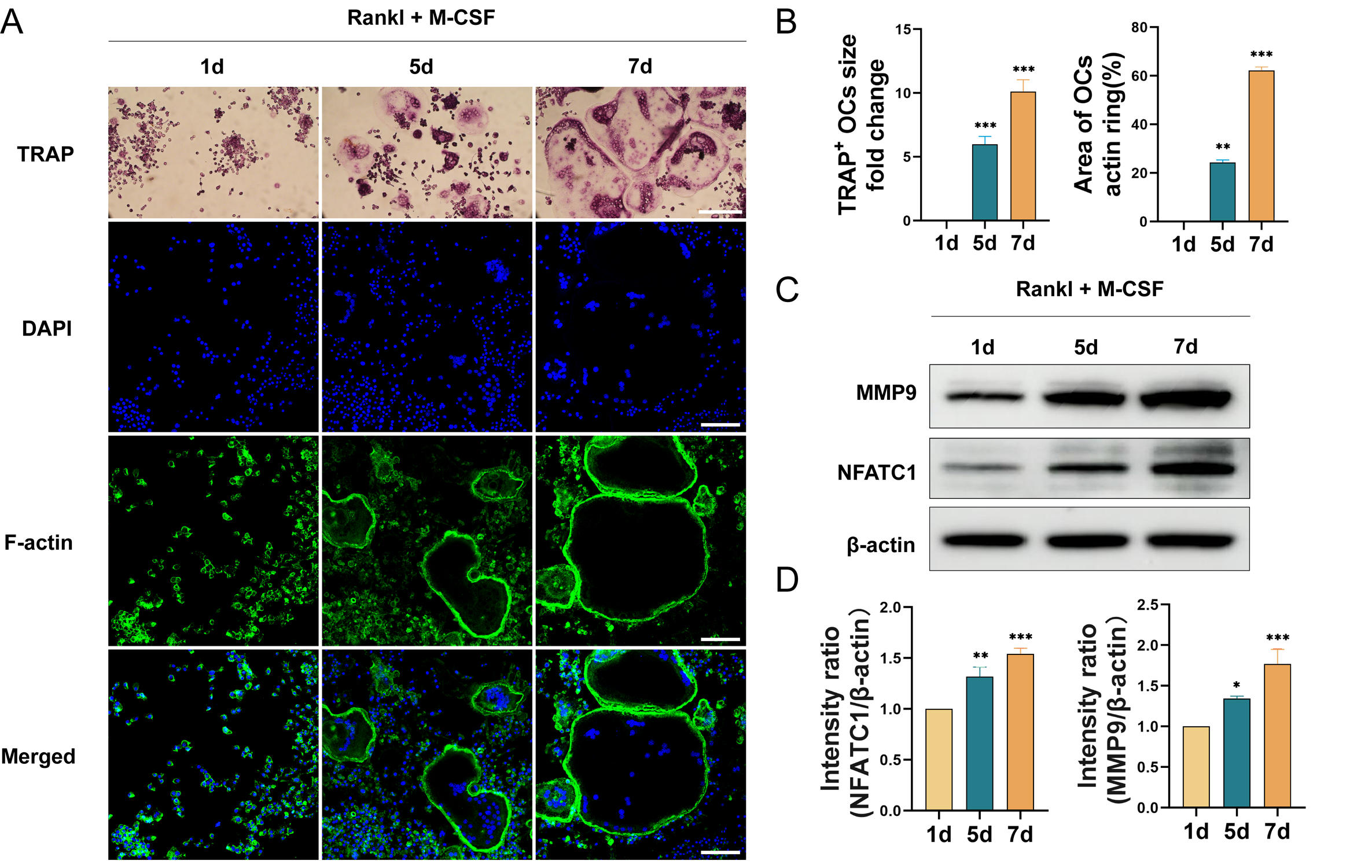

Supplement: Supplementary file 3 — Figure S3: Establishment of osteoclast differentiation model. (A) Bone marrow macrophages (BMMs) were induced with RANKL and M‐CSF for different periods. TRAP staining was used to visualise osteoclast formation and laser confocal microscopy was used to observe the formation of actin rings in osteoclasts (n = 3). Scale bar: 100 μm. (B) Quantitative analysis of size changes in TRAP‐positive osteoclasts and the area of actin ring formation in osteoclasts. (C) Western blotting analysis of MMP9 and NFATC1 protein expression in BMMs at different time points after induction (n = 3). (D) Quantitative analysis of ITGB2, ITGB1, ITGA2, and Total‐Rac1 expression during osteoclastogenesis compared with the 1d group. *Error bars represent the mean ± standard deviation; statistical significance: ***p < 0.001, **p < 0.01, p < 0.05, ns, no significant difference. Comparisons between two groups were conducted using Student's t‐test. [file CPR-59-e70107-s005.tif]

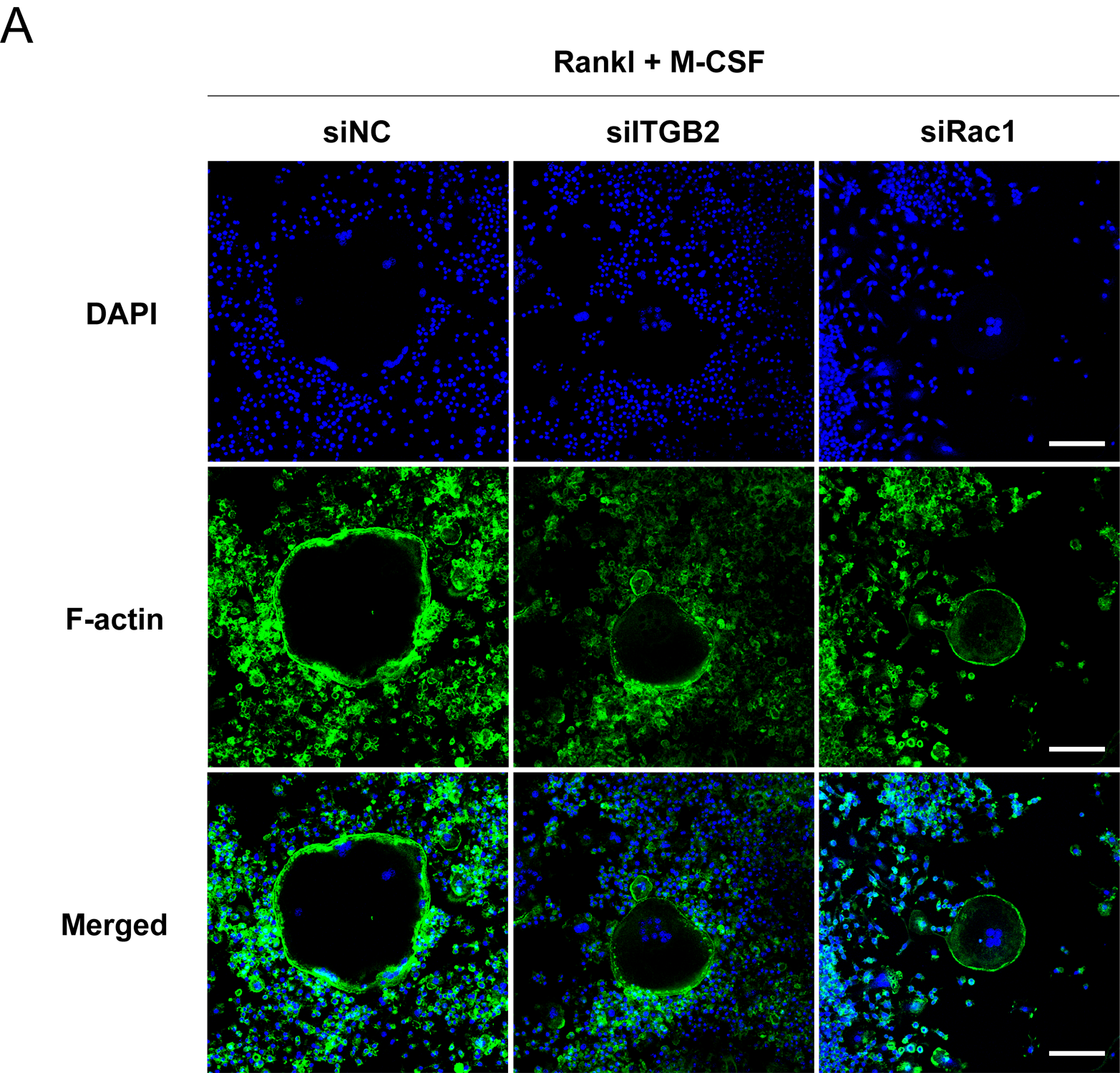

Supplement: Supplementary file 4 — Figure S4: ITGB2 and Rac1 deficiency inhibits actin ring formation and leads to osteoclast differentiation defects. (A) BMMs and RAW264.7 cells were treated under different conditions (siNC, siITGB2, and siRac1), followed by 7 days of induction with RANKL and M‐CSF. Immunofluorescence staining was performed to assess the effects of these treatments on osteoclast differentiation. The upper panel shows DAPI staining of the nuclei (blue), the middle panel shows F‐actin distribution (green), and the bottom panel shows a merged image of the nuclei and cytoskeleton. Scale bar: 100 μm. [file CPR-59-e70107-s006.tif]

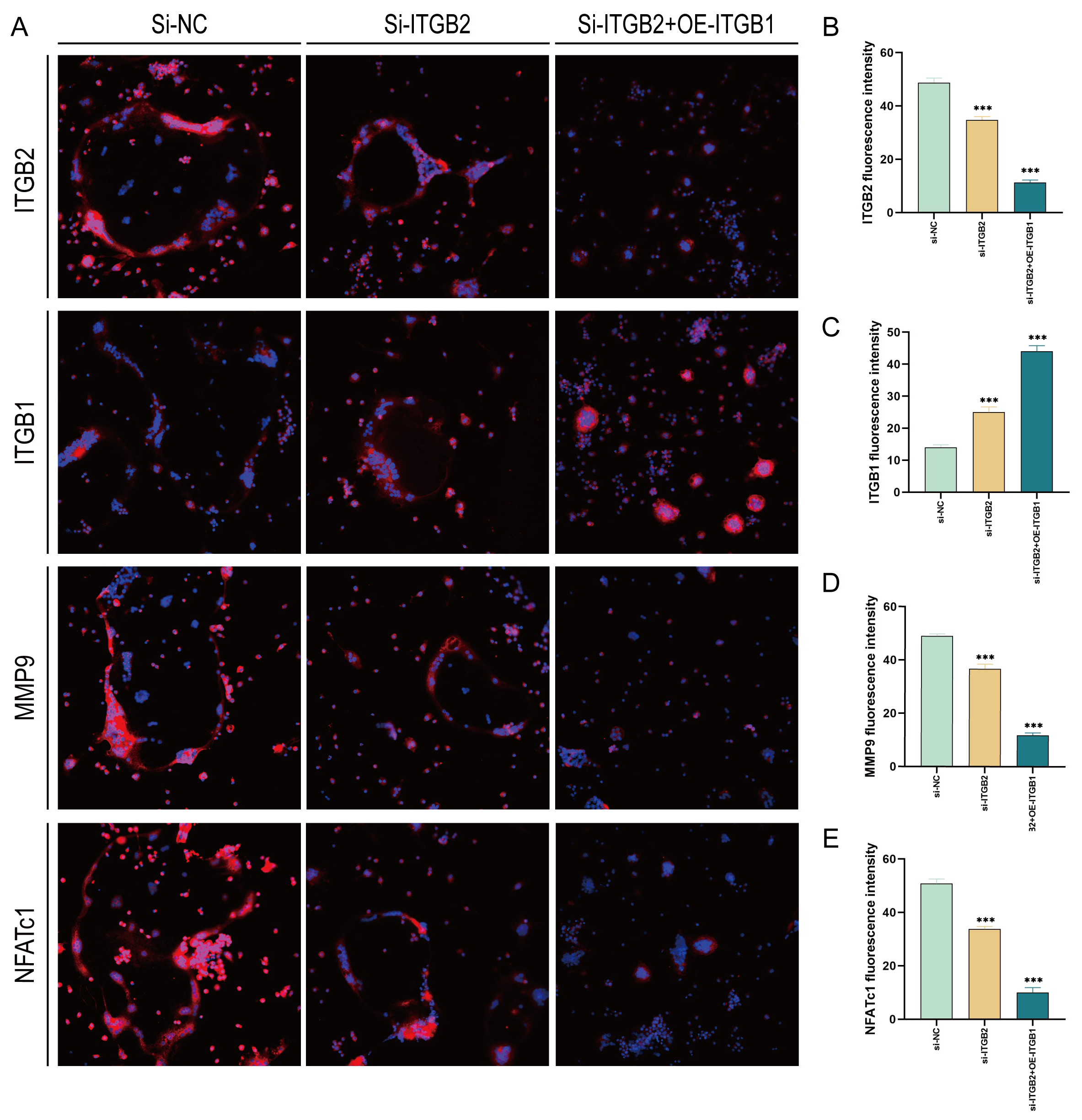

Supplement: Supplementary file 5 — Figure S5: Overexpression of ITGB1 further reduces ITGB2 expression and potentiates the inhibitory effect of ITGB2 deficiency on osteoclast differentiation. (A) Bone marrow‐derived macrophages (BMMs) were treated under different conditions (siNC, siITGB2, and siITGB2 + OE‐ITGB1), followed by induction with RANKL and M‐CSF for 7 days. Immunofluorescence staining was performed to evaluate the effects of these treatments on osteoclast differentiation and protein expression during differentiation. Nuclei were stained with DAPI (blue), and protein distributions are shown in red. Scale bar: 100 μm. OE, overexpression. Quantitative analysis of fluorescence intensities for (B) ITGB2, (C) ITGB1, (D) MMP9, and (E) NFATc1 proteins. *Error bars represent the mean ± standard deviation; statistical significance: ***p < 0.001, **p < 0.01, p < 0.05, ns, no significant difference. Comparisons between two groups were conducted using Student's t‐test. [file CPR-59-e70107-s002.tif]

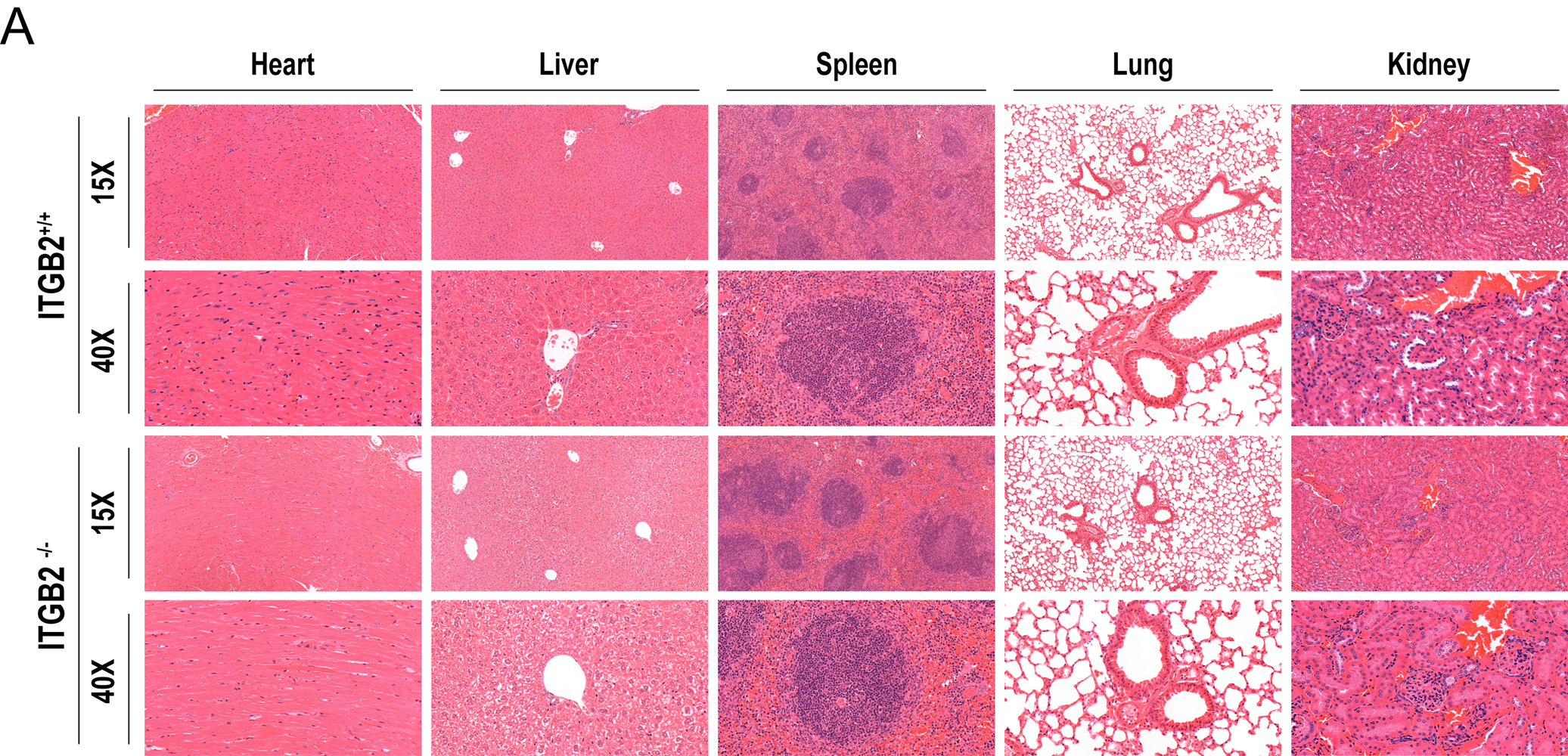

Supplement: Supplementary file 6 — Figure S6: Effect of ITGB2 Knockout on the heart, liver, spleen, lungs, and kidney structure. (A) Haematoxylin and eosin (H&E) staining was performed on the heart, liver, spleen, lungs, and kidneys before DMM surgery to assess histological features. The results showed no significant changes in the basic structural characteristics compared to those of the sham surgery group. [file CPR-59-e70107-s003.tif]
