# Supplementary material for: Mechanism of ITGB2 in Osteoclast Differentiation in Osteoarthritis
Source: Cell Prolif. 2025 Jul 29;59(3):e70107. doi: 10.1111/cpr.70107 (PMC12961538; doi:10.1111/cpr.70107)
Supplement: Supplementary file 7 — Table S1: Real‐time PCR primers. [file CPR-59-e70107-s010.docx]

**Table 1**

Real-time PCR primers

| Gene symbol (Mus) | Primer sequences (5' - 3') |
| --- | --- |
| ITGB2 | Forward: TGCGTCCTCTCTCAGGAGTG |
|  | Reverse: GGTCCATGATGTCGTCAGCC |
| ITGB1 | Forward: CCTACTTCTGCACGATGTGATGT |
|  | Reverse: CCTTTGCTACGGTTGGTTACATT |
| ITGA2 | Forward: TTGGAACGGGACTTTCGCAT |
|  | Reverse: GGTACTTCGGCTTTCTCATCA |
