# Supplementary material for: Mechanism of ITGB2 in Osteoclast Differentiation in Osteoarthritis
Source: Cell Prolif. 2025 Jul 29;59(3):e70107. doi: 10.1111/cpr.70107 (PMC12961538; doi:10.1111/cpr.70107)
Supplement: Supplementary file 9 — Table S3: Summary of antibodies employed in the study. [file CPR-59-e70107-s008.docx]

**Table 3**

Summary of Antibodies Employed in the Study

| Gene symbol (Mus) | Catalog Number | Supplier | Dilution |
| --- | --- | --- | --- |
| ITGB2 | ab307406 | Abcam | 1:1,000 |
| ITGB1 | sc-374429 | Santa Cruz | 1:1,000 |
| ITGA2 | ab181548 | Abcam | 1:1,000 |
| Rac1 | sc-514583 | Santa Cruz | 1:1,000 |
| CTSK | sc-48353 | Santa Cruz | 1:500 |
| ACP5 | sc-376875 | Santa Cruz | 1:500 |
| MMP9 | 10375-2-AP | Proteintech | 1:1,000 |
| NFATC1 | sc-7294X | Santa Cruz | 1:1,000 |
| β-actin | 20536-1-AP | proteintech | 1:1,0000 |
