# Supplementary material for: Mechanism of ITGB2 in Osteoclast Differentiation in Osteoarthritis
Source: Cell Prolif. 2025 Jul 29;59(3):e70107. doi: 10.1111/cpr.70107 (PMC12961538; doi:10.1111/cpr.70107)
Supplement: Supplementary file 10 — Table S4: Gene identification primers. [file CPR-59-e70107-s007.docx]

**Table 4**

Gene identification primers.

| Gene symbol (Mus) | Primer sequences (5' - 3') |
| --- | --- |
| ITGB2 | Forward: GCAUAUGUGACGAAGGCUATT |
|  | Reverse: UAGCCUUCGUCACAUAUGCTT |
| Rac1 | Forward: CCCACCGTCTTTGACAACTATTCTG |
|  | Reverse:AGACAATAGTTGTCAAAGACGGTGG |
